# The antipsychotic-like effects in rodents of the positive allosteric modulator Lu AF21934 involve 5-HT_1A_ receptor signaling: mechanistic studies

**DOI:** 10.1007/s00213-014-3657-4

**Published:** 2014-07-11

**Authors:** Joanna M. Wierońska, Anna Sławińska, Magdalena Łasoń-Tyburkiewicz, Piotr Gruca, Mariusz Papp, Stevin H. Zorn, Darío Doller, Natalia Kłeczek, Karolina Noworyta-Sokołowska, Krystyna Gołembiowska, Andrzej Pilc

**Affiliations:** 1 Institute of Pharmacology, Polish Academy of Sciences, Smętna Str. 12, 31-343 Kraków, Poland; 2 Lundbeck Research USA, 215 College Road, Paramus, NJ 07652 USA; 3 Jagiellonian University, Collegium Medicum, Michałowskiego 12, 31-126 Kraków, Poland

**Keywords:** Glutamate, Antipsychotic, Behavior, Excitatory amino acid, Glutamate receptor, Metabotropic glutamate receptor, MK-801, Serotonin receptor

## Abstract

**Rationale:**

Diverse preclinical studies suggest the potential therapeutic utility of the modulation of the glutamatergic system in brain via metabotropic glutamate (mGlu) receptors. Lu AF21934, a positive allosteric modulator of the mGlu4 receptor, was previously shown to reverse behavioral phenotypes in animal models thought to mimic positive, negative, and cognitive symptoms of schizophrenia.

**Objectives:**

To begin elucidating the brain circuitry involved in mGlu4 receptor pharmacology and add mechanistic support to Lu AF21934-induced phenotypic responses, the potential involvement of 5-HT_1A_ receptors in these antipsychotic-like effects was explored. The tests used were the following: MK-801-induced hyperactivity and 2,5-dimethoxy-4-iodoamphetamine (DOI)-induced head twitches in mice, for positive symptoms; MK-801-induced disruptions of social interactions for negative symptoms; and novel object recognition and spatial delayed alteration test for cognitive symptoms. The microdialysis studies in which the effect of Lu AF21934 on MK-801-induced dopamine and serotonin release was investigated.

**Results:**

The effects caused by Lu AF2193 were inhibited by administration of the selective 5-HT_1A_ receptor antagonist WAY100635 (0.1 mg/kg). That inhibition was observed across all models used. Moreover, the concomitant administration of sub-effective doses of Lu AF21934 and a sub-effective dose of the selective 5-HT_1A_ receptor agonist tool compound (*R*)-(+)-8-hydroxy-DPAT hydrobromide (0.01 mg/kg) induced a clear antipsychotic-like effect in all the procedures used. Lu AF21934 (5 mg/kg) also inhibited MK-801-induced increase in dopamine and 5-HT release.

**Conclusions:**

The actions of Lu AF21934 are 5-HT_1A_ receptor-dependent. Activation of the mGlu4 receptor may be a promising mechanism for the development of novel antipsychotic drugs, efficacious toward positive, negative, and cognitive symptoms.

**Electronic supplementary material:**

The online version of this article (doi:10.1007/s00213-014-3657-4) contains supplementary material, which is available to authorized users.

## Introduction

The glutamatergic system represents the basic excitatory neurotransmission mechanism in the brain. Clinically, the administration of *N*-methyl-D-aspartate (NMDA) antagonists (e.g., MK-801, PCP) leads to the arousal of the full spectrum of schizophrenic symptoms, including positive, negative, and cognitive disturbances (Krystal et al. 2002; Moghaddam and Jackson 2003). In contrast, drugs known as psychotomimetics (dopaminergic and serotonergic agonists) evoke only positive symptoms of schizophrenia. These observations support the involvement of the glutamatergic system in the pathophysiology of psychotic disorders (Bickel and Javitt 2009; Javitt and Zukin 1991; Javitt et al. 2004).

According to the glutamatergic hypothesis, blockade of NMDA receptors expressed on GABAergic neurons leads to the inhibition of GABAergic control over pyramidal neurons in the thalamocortical pathway. In turn, this would lead to abnormal glutamate release and to the overactivation of cortical and subcortical structures (Krystal et al. 2002; Moghaddam and Jackson 2003; Conn et al. 2009). Therefore, the reharmonization of such enhanced release may lead to the normalization of glutamate efflux and to the reversal of the symptoms of schizophrenia (Conn et al. 2009).

GABAergic and glutamatergic metabotropic receptors have been proposed as relevant targets involved in the regulation of glutamate release (Conn and Pin 1997; Pałucha-Poniewiera et al. 2008; Pin and Duvoisin 1995; Schoepp and Marek 2002; Wierońska et al. 2011). Among these, the presynaptic metabotropic glutamate mGlu_2/3_ and mGlu_4_ receptors appear optimal targets for potential antipsychotic treatment. All of these are negatively linked to adenylyl cyclase activity, and their activation inhibits glutamate release (Pin and Duvoisin 1995). Moreover, agonist and positive allosteric modulator (PAM) tool compounds such as mGlu_2/3_ activators (LY354740, LY379268, CIBeS, BINA) (Cartmell et al. 2000; Galici et al. 2005; Fell et al. 2008), and mGlu_4_ activators (ACPT-I, LSP1-2111, Lu AF21934, and Lu AF32615) were shown to be active in animal models of schizophrenia-like behaviors (Pałucha-Poniewiera et al. 2008; Sławińska et al. 2013). A few mGlu_2/3_ agonists (most recently pomaglumetad methionil or LY2140023), and an mGlu_2_ PAM (ADX71149 or JNJ-40411813) have also reached clinical testing, with mixed results (Patil et al. 2007; Kinon et al. 2011; Hopkins 2013).

Herein, we report follow-up investigations on the mechanism of action of Lu AF21934, focusing on the functional interaction between the mGlu_4_ and 5-hydroxytryptamine (HT)_1A_ receptors.

The 5-HT_1A_ receptors seem to be of special importance in schizophrenia, as the increased 5-HT_1A_ receptor level was observed in the postmortem study of the cerebral cortex and hippocampus of schizophrenic patients (Burnet et al. 1996; Simpson et al. 1996). Moreover, the majority of novel atypical neuroleptics with good antipsychotic efficacy (ziprasidone and lurasidone) are partial agonists of 5-HT_1A_ receptors (Schmidt et al. 2001; Sprouse et al. 1999; Ichikawa et al. 2002; Hagiwara et al. 2008). And finally, the 5-HT_1A_ agonist, 8-OH-DPAT, at the low doses, induced antipsychotic-like effects in animal models (Bubeníková-Valesová et al. 2007; Boulay et al. 2004).

These studies were motivated by earlier work showing that the actions of the mGlu_4_ receptor agonist LSP1-2111 is 5-HT_1A_ receptor-dependent. This interdependence was shown in a number of preclinical models thought to represent positive, negative, and cognitive symptoms of schizophrenia. We used the antagonist WAY100635 and the agonist (*R*)-(+)-8-hydroxy-DPAT as 5-HT_1A_ receptor-selective tool compounds, concomitantly administered with Lu AF21934. In addition, Lu AF21934 was also co-administered with lurasidone, a recently approved atypical antipsychotic with nonselective CNS effects, including potent 5-HT_1A_ receptor agonism.

## Materials and methods

### Animals and housing

Male Albino Swiss (20–25 g) mice were used to assess MK-801-induced hyperlocomotion, and 2,5-dimethoxy-4-iodoamphetamine (DOI)-induced head twitches. Male Wistar rats weighing 250–300 g were used in the social interaction, novel object recognition, and spatial delayed alternation tests. The choice of animal species was based on our preliminary experiments, as well as on literature data, and was dictated by the optimization of the method to obtain credible results and minimization of the costs of experiments. The animals were kept under a 12:12 light-dark cycle at a room temperature of 19–21 °C, with free access to food and water. Each experimental group consisted of eight to ten animals, and the animals were used only once in each test. All the compounds were used in a volume of 10 ml/kg when given to mice and 1 ml/kg when injected to rats. All behavioral measurements were made by an observer blinded to the treatment. All procedures were conducted according to the guidelines of the National Institutes of Health Animal Care and Use Committee and were approved by the Ethics Committee of the Institute of Pharmacology, Polish Academy of Sciences in Krakow and Lundbeck Research USA.

### Drugs

The following drugs were used: Lu AF21934 (mGlu4 receptor PAM) synthesized through Lundbeck Research USA, and characterized using H-1 and C-13 nuclear magnetic resonance spectroscopy, high performance liquid chromatography (HPLC)/mass-spectrometry methods, and X-ray crystallography. The compound was dosed as a suspension in 20 % (2-hydropropyl)-β-cyclodextrin and was administered subcutaneously (s.c.) 60 min before the tests. The administration schedule of Lu AF21934 was planned according to our previous studies on anxiety and psychosis (Sławińska et al. 2013). MK-801 (0.35, Sigma-Aldrich, St. Louis, USA) was dissolved in 0.9 % NaCl, and the doses were selected consistently with our previous work (Pałucha-Poniewiera et al. 2008; Wierońska et al. 2012, 2013a, b) and that of others (Geyer and Ellenbroek 2003; Leite et al. 2008; Satow et al. 2009). WAY100635 and (*R*)-(+)-8-hydroxy-DPAT (Tocris Bioscience, Bristol, United Kingdom) were dissolved in 0.9 % saline and were administered according to our previous studies and also those made by others (Wierońska et al. 2013a, b; Wedzony et al. 2000). Lurasidone hydrochloride (Ontario Chemicals, 7A-291 Woodlawn Road, Guelph, ON, N1H7L6, Canada) was dissolved in 0.5 % methylcellulose (Horiguchi et al. 2012).

### Locomotor activity of habituated mice

The locomotor activity was recorded individually for each animal in OPTO-M3 locomotor activity cages (Columbus Instrument) linked online to a compatible PC. Each cage (13 cm × 23 cm × 15 cm) was surrounded with an array of photocell beams. Interruptions of these photobeams resulted in horizontal activity defined as ambulation scores. Mice were placed separately into activity cages for an acclimatization period of 30 min, and then, they were injected s.c. with Lu AF21934, WAY100635, (*R*)-(+)-8-hydroxy-DPAT and their combinations (time and doses of administration similar as described below for MK-801-induced hyperactivity). From this point on, the ambulation scores were measured for 60 min.

### MK-801-induced hyperactivity

The locomotor activity was recorded for each animal in locomotor activity cages (according to Rorick-Kehn et al. 2007a, b), with small modifications used in our previous studies (Pałucha-Poniewiera et al. 2008; Wierońska et al. 2012, 2013a, b). The mice were placed individually into actometers for an acclimatization period of 30 min; then they were administered Lu AF21934 (60 min before MK-801, s.c), WAY100635 (45 min before MK-801, intraperitoneally (i.p)) and (*R*)-(+)-8-hydroxy-DPAT (15 min before MK-801, s.c), or vehicle and placed again in the same cages. After the proper time, all of the mice were administered intraperitoneally with MK-801 at a dose of 0.35 mg/kg and once again returned to the same cage. From then on, the ambulation scores were counted for 60 min. All groups were compared with the MK-801 control group. The experiment also included a control group not treated with MK-801.

### Head twitch test

The experiment was performed according to Pałucha-Poniewiera et al. (2008) and Wieronska et al. (2012, 2013a, b). In order to habituate mice to the experimental environment, each animal was transferred to a 12 (diameter) × 20 cm (height) glass cage, lined with sawdust, 30 min before the treatment. The head twitches of the mice were induced by DOI (2.5 mg/kg, i.p.). Immediately after the treatment, the number of head twitches was counted during a 20-min session. Lu AF21934, WAY100635 and (*R*)-(+)-8-hydroxy-DPAT were dosed 60, 45, and 15 min prior to the DOI administration, respectively.

### MK-801-induced deficits in social interaction test in rats

Social interaction tests were performed according to the method described by Satow et al. 2009, using a circle made of wood, 90 cm in diameter divided into 10 × 10 cm squares by faint yellow lines. Each social interaction test between two rats was carried out during the light phase of the light/dark cycle. Rats were selected from separate housing cages to make a part for the study. The body weights of the paired rats were matched within 20 g of variance. All rats were placed in an experimental room, and the study was conducted 3.5 h after the subcutaneous administration of MK-801 at a dose of 0.1 mg/kg, s.c. Lu AF21934, WAY100635, and (*R*)-(+)-8-hydroxy-DPAT were dosed 60, 45, and 15 min prior to the MK-801 administration, respectively.

The test box was wiped clean between each trial. Social interaction between two rats was determined as the total time spent participating in social behavior such as sniffing, genital investigation, chasing, and fighting each other. The total number of social episodes was also measured. In addition, control experiments in animals not receiving MK-801 were also conducted, in order to establish if the drugs had any influence on social behavior when given alone.

### Spatial delayed alternation test in rats

The animals, deprived of water overnight, were trained and tested in four wooden T-mazes, which consisted of white and black end-arms (33 × 22 × 25 cm) and a gray starting arm (15 × 20 × 25 cm). The end-arms were equipped with a spout bottle located 9 cm above the floor and containing a 10 % sucrose solution. The three arms were separated from each other by guillotine doors.

### Adaptation

On the first 3 days, the animals were allowed freely to explore the whole T-maze for 10 min. On the next 2 days, they were confined to either of the two end-arms and allowed to drink the sucrose solution there for 10 min twice daily.

### Training

On the next 2 weeks, the animals received once daily training sessions. Each session consisted of one forced trial (i.e., when one of the end-arm was closed) followed by ten free choice trials. For each free choice trial, the animals were placed in the starting arm, the guillotine doors were raised, and when the rat entered one of the end-arm, the guillotine door was closed, and the rat was allowed to drink the sucrose solution there for 5 s. Then, the rat was gently returned to the starting arm, where it stayed for 10 s (delayed interval). After that time, the guillotine door was raised, and the rat was allowed to enter the end-arms. If the end-arm chosen was opposite to that visited on the previous trial (a correct response), the sucrose solution was provided, and the drinking was allowed for 5 s. If the end-arm chosen was the same as on the previous trial, an incorrect response was scored, and the animal gently returned to the starting arm for 10 s (delayed interval). This training was continued until the animals reach performance criterion, which was defined as at least seven correct responses in ten trials for two consecutive daily sessions.

### Testing

The animals were injected with a drug, and the above procedure was repeated; the rat was placed in the starting arm, the guillotine doors raised, and the rat was allowed to enter the end-arms. If it chose the correct end-arm (i.e., opposite to that visited on the previous trial), the sucrose solution was provided; 5 s later, the rat was returned to the starting arm for 10 s (delayed interval). If the end-arm selected was incorrect (i.e., the same as on the previous trial), the rat was returned to the starting arm for 10 s. Such testing sessions were carried out once a week and were preceded by two daily training sessions.

### Novel object recognition (NOR)

The method was adapted from Horiguchi et al (2011a, b) and Dere et al. (2007). The animals were trained and tested in a black wooden circular open field (100 cm in diameter, 35 cm high) with the floor divided into 20-cm square sections. The open field was in a dark room illuminated only by a 25 W bulb. On the first day (adaptation), the animals were allowed to explore the open field for 10 min. On the next day (training, T_1_) the animals were administered with the tested drugs, placed in the apparatus, and allowed to explore two identical objects (cylinder-shaped objects with walls painted white, 7 cm in diameter, 11 cm high) for the time required to complete 15 s of exploration of either object. For the retention trial (T_2_) conducted 1 h later, one of the objects presented in T_1_ was replaced with a novel object (a prism-shaped object with walls painted black, 5 cm wide, 14 cm high). The rats were returned to the open field for 5 min, and the duration of exploration (i.e., sitting in close proximity to the objects, sniffing, or touching them) of each object was measured separately by a trained observer. All drugs were administered before the training (T_1_) session. MK-801 (0.1 mg/kg, s.c) was given 30 min before the session. Lu AF21934, WAY100635, and (*R*)-(+)-8-hydroxy-DPAT were dosed 60, 45, and 15 min prior to the MK-801 administration, respectively. All injections were given at a volume of 1 ml/kg of the body weight. The treatment groups included eight animals.

## In vivo microdialysis

Rats were anesthetized with ketamine (75 mg/kg intramuscular (i.m.)) and xylazine (10 mg/kg i.m.) and placed in a stereotaxic apparatus (David Kopf Instruments, Tujunga, CA, USA). Their skulls will be exposed, and small holes were drilled for the insertion of microdialysis probes into the brain structures using appropriate coordinates (Paxinos and Watson 1982). Vertical microdialysis probes were constructed as described in detail elsewhere (Golembiowska et al. 2012). Following 24 h of the surgery probe, inlets were connected to a syringe pump (BAS, IN, USA) which delivered an artificial CSF composed of [mM]: NaCl 147, KCl 4.0, MgCl_2_ 1.0, CaCl_2_ 2.2; pH = 7.4 at a flow rate of 2.0 μl/min. Baseline samples were collected every 20 min for 2 h after the washout period to obtain a stable extracellular neurotransmitter level. Then, tested drugs were injected, and subsequent fractions of dialysates were collected for 3 h. At the end of the experiment, the rats were sacrificed, and their brains were examined histologically to validate probe placement.

## Analytical procedure

DA and 5-HT were analyzed by HPLC with coulochemical detection. Chromatography was performed using an Ultimate 3000 System (Dionex, USA), coulochemical detector Coulochem III (model 5300, ESA, USA) with 5020 guard cell, 5014B microdialysis cell, and Hypersil Gold-C_18_ analytical column (3 × 100 mm). The mobile phase will be composed of 0.1 M potassium phosphate buffer adjusted to pH = 3.6, 0.5 mM EDTA, 16 mg/l 1-octanesulfonic acid sodium salt, and 2 %. The flow rate during analysis was set at 0.7 ml/min. The applied potential of a guard cell was +600 mV, while those of microdialysis cells were the following: *E*_1_ = −50 mV, *E*_2_ = +300 mV with a sensitivity set at 50 nA/V. The chromatographic data was processed by Chromeleon v. 6.80 (Dionex, USA) software run on a PC computer.

### Statistical analysis

The data are presented as the means ± SEM. Statistical analysis of the data was performed using the Graph Pad Prism ver.5 and Statistica 10 package (StatSoft Inc., OK, USA). Two-way ANOVA, followed by Newman-Keuls post hoc comparison test, was used in the interaction studies. The *P* value of at least *P* < 0.05 was considered as statistically significant. In vivo microdialysis: All obtained data were given as a percent of basal level assumed as 100 %. The statistical significance was calculated using repeated-measures ANOVA, followed by Tukey’s post hoc test. The results were considered statistically significant when *P* < 0.05.

## Results

### The effects of the combined administration of WAY100635 and Lu AF21934 on MK-801-induced hyperactivity in mice

**Fig. 1:**
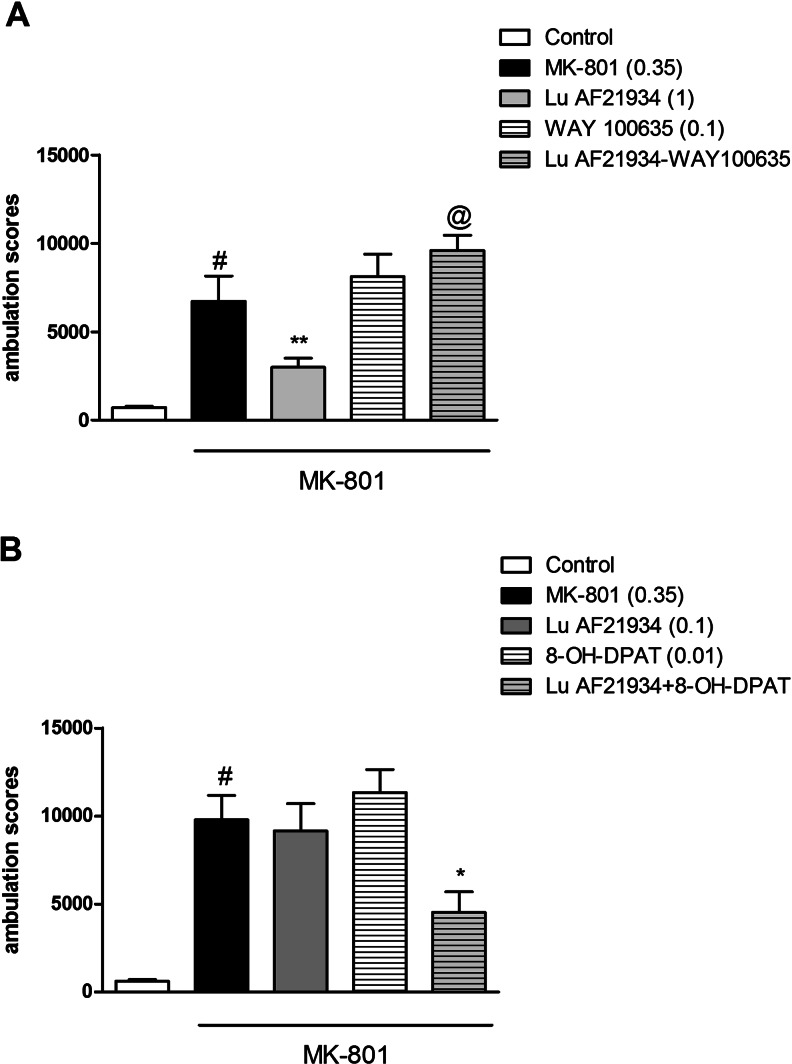
Effects on MK-801-induced hyperactivity. The combined administration of Lu AF21934 (1 mg/kg) with WAY100635 (0.1 mg/kg) (**a**), and the co-administration of Lu AF21934 (0.1) with 8-OH-DPAT (0.01) (**b**) are presented. Data are presented as means ± SEM. Doses in milligrams per kilogram are indicated in *parentheses*. ^#^ *P* < 0.01 versus control, **P < 0.01 versus MK-801-treated group, @*P* < 0.05 versus Lu AF21934-treated group

Lu AF21934 administered at a dose of 1 mg/kg, s.c, induced a clear antipsychotic-like effect, reversing MK-801-induced hyperactivity (*P* < 0.009). WAY100635, administered at a dose of 0.1 mg/kg, i.p., did not have any effect on its own. Co-administration of WAY100635 with Lu AF21934 (1 mg/kg) resulted in the inhibition of Lu AF21934-induced effect in the hyperactivity test. The two-way ANOVA main effects analysis revealed the significant effect of Lu AF21934 × WAY100635 interaction [F_(1.29)_ = 8.02; *P* < 0.008] (Fig. 1a).

### The effects of the combined administration of sub-effective doses of (*R*)-(+)-8-hydroxy-DPAT hydrobromide and Lu AF21934 on MK-801-induced hyperactivity in mice

Lu AF21934 was administered at a dose of 0.1 mg/kg, and (*R*)-(+)-8-hydroxy-DPAT hydrobromide was given at a dose of 0.01 mg/kg. Neither compound had any effects when dosed separately. Concomitant administration of the sub-effective doses of 5-HT_1A_ receptor agonist and the mGlu_4_ receptor PAM-induced clear reversal of hyperactivity. Two-way ANOVA main effects analysis revealed the significant effect of (*R*)-(+)-8-hydroxy-DPAT and Lu AF21934 interaction [F_(1.29)_ = 5.11, *P* < 0.03]. Post hoc Newman-Keuls analysis revealed the significant effect of (*R*)-(+)-8-hydroxy-DPAT × Lu AF21934 when compared to the MK-801-treated animals, *P* < 0.02 (Fig. 1b). The attenuation of the MK-801-induced hyperlocomotion reached 46 %.

### The effects of the combined administration of WAY100635 and Lu AF21934 on DOI-induced head twitches in mice

**Fig. 2:**
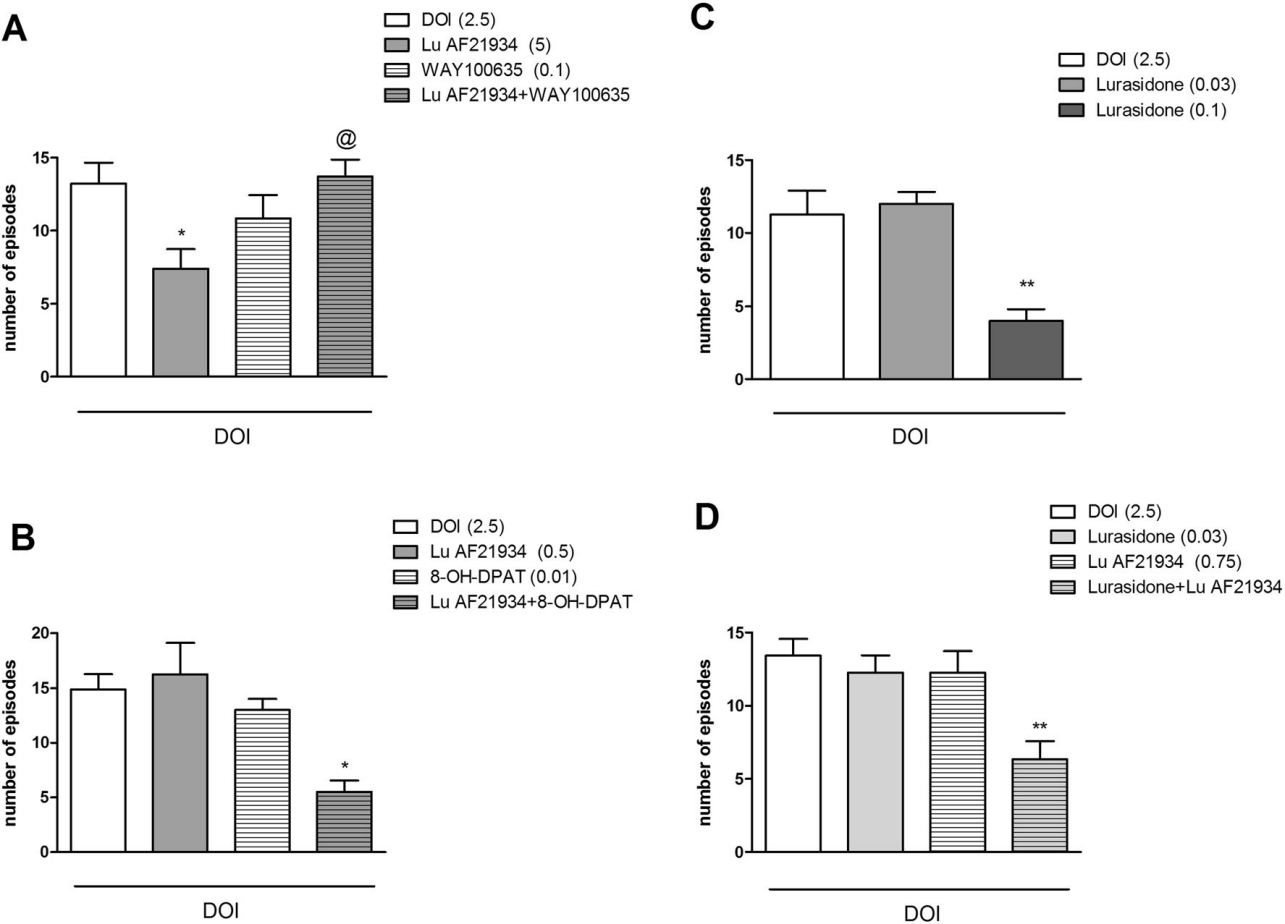
Effects on DOI-induced head twitches. The combined administration of Lu AF21934 (5 mg/kg) with WAY100635 (0.1 mg/kg) (**a**), and the co-administration of Lu AF21934 (0.5 mg/kg) with 8-OH-DPAT (0.01 mg/kg) (**b**), as well as the administration of lurasidone (0.1 and 0.03 mg/kg) (**c**), and the effect of the lurasidone + Lu AF21934 combination (**d**) are presented. Data are presented as means ± SEM. Doses in milligrams per kilogram are indicated in *parentheses*. *P < 0.05 and **P < 0.01 versus DOI-treated group, @*P* < 0.05 versus Lu AF21934-treated group

Lu AF21934 administered at a dose of 2 mg/kg significantly inhibited the number of DOI-induced head twitches (*P* < 0.01). WAY100635, administered at a dose of 0.1 mg/kg did not have any effect on its own. Co-administration of Lu AF21934 and WAY100635 resulted in the inhibition of Lu AF21934-induced effect [F_(1.26)_ = 9.46, *P* < 0.004]. Post hoc Newman-Keuls analysis revealed a significant Lu AF21934 × WAY100635 interaction when compared to Lu AF21934 treated group, *P* < 0.01 (Fig. 2a).

### The effect of the combined administration of (*R*)-(+)-8-hydroxy-DPAT hydrobromide and a sub-effective dose of Lu AF21934 on DOI-induced head twitches in mice

Lu AF21934 was administered at a dose of 1 mg/kg, and (*R*)-(+)-8-hydroxy-DPAT hydrobromide was given at a dose of 0.01 mg/kg. Neither drug had effects when dosed alone. Concomitant administration of sub-effective doses of 5-HT_1A_ receptor agonist and mGlu_4_ receptor PAM-induced clear reduction in the number of DOI-induced head twitches. The two-way ANOVA main effects analysis revealed the significant effect of Lu AF21934 × (*R*)-(+)-8-hydroxy-DPAT hydrobromide interaction [F_(1.26)_ = 8.25, *P* < 0.007]. Post hoc Newman-Keuls analysis revealed the significant effect of Lu AF21934 × (*R*)-(+)-8-hydroxy-DPAT hydrobromide when compared to the DOI-treated animals, *P* < 0.0003 (Fig. 2b). The abolishment of the DOI-induced head twitches reached 37 %.

### The effect of the combined administration of lurasidone and a sub-effective dose of Lu AF21934 on DOI-induced head twitches in mice

Lurasidone hydrochloride abolished the DOI-induced head twitches at a dose of 0.1 mg/kg, when injected 30 min prior to testing [F_(2.19)_ = 13.45; *P* < 0.0002]. The dose of 0.03 mg/kg was ineffective. The concomitant administration of ineffective doses of Lu AF21934 (1 mg/kg), and lurasidone (0.03 mg/kg) induced clear antipsychotic-like effect in the DOI-induced head twitch test (Fig. 2c). The two-way ANOVA main effect analysis revealed the significant effect of Lu AF21934 × lurasidone interaction [F_(1.27)_ = 7.31, *P* < 0.02]. Post hoc Newman-Keuls analysis revealed the significant effect of Lu AF21934 × lurasidone when compared to the DOI-treated animals, *P* < 0.0002 (Fig. 2d).

### The effect of the combined administration of WAY100635 and Lu AF21934 in the social interaction test in rats

**Fig. 3:**
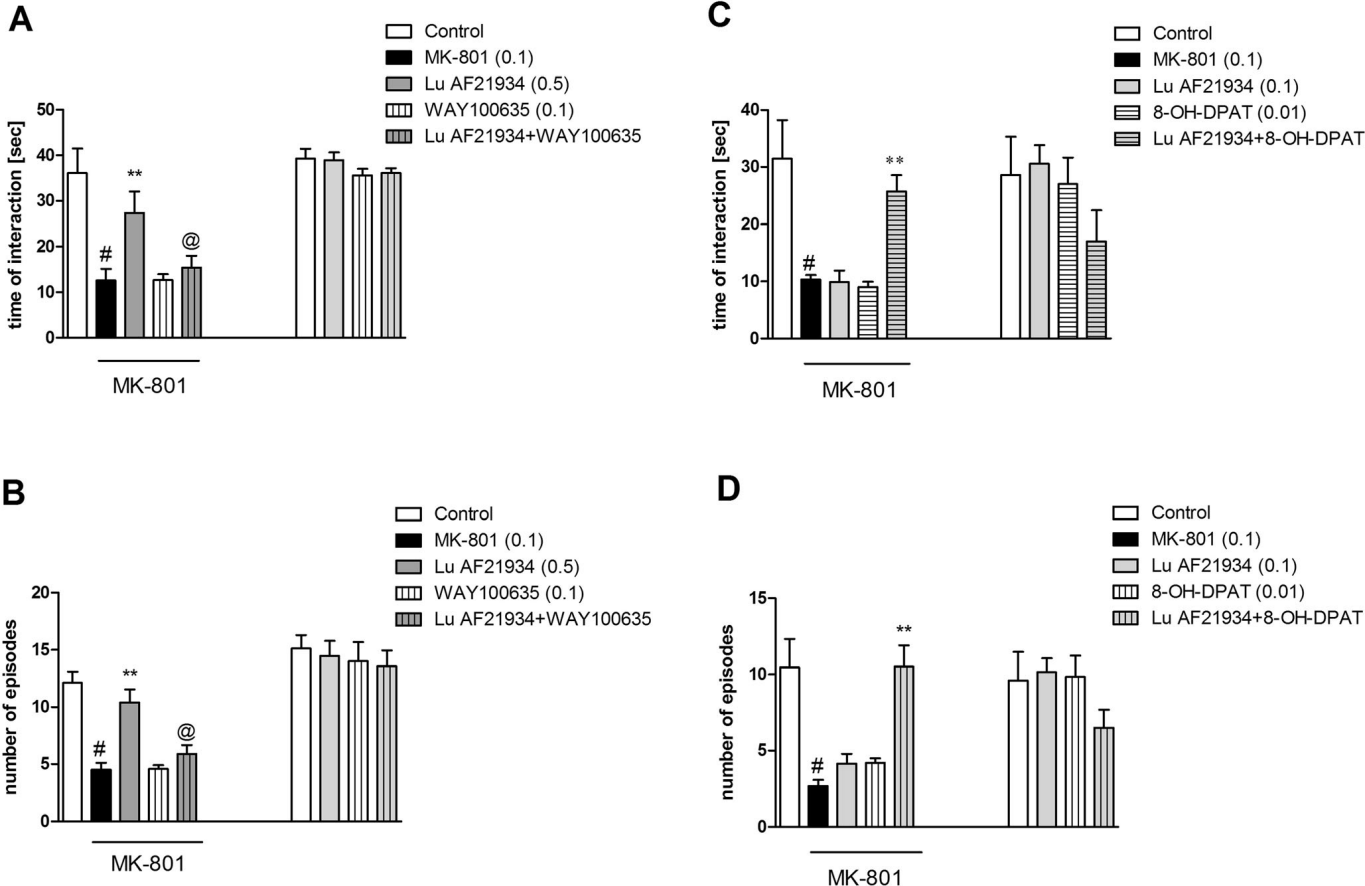
Effects on MK-801-induced deficits in social interaction. Number of episodes of social contacts and time of social interactions were measured. Effects of combined administration of Lu AF21934 and WAY100635 in MK-801-treated rats and control experiments (**a**, **b**). Effects of combined administration of Lu AF21934 and 8-OH-DPAT in MK-801-treated rats and control experiments (**c**, **d**). Data are presented as means ± SEM. Doses in milligrams per kilogram are indicated in *parentheses*. At least #*P* < 0.05 versus controls, *P < 0.05 versus MK-801-treated group and @*P* < 0.05 versus Lu AF21934-treated group

Lu AF21934 was given at a dose of 0.5 mg/kg, 60 min before the test. The compound reversed social deficits induced by MK-801 administration (number of social contacts and the total duration of contacts, *P* < 0.0002). WAY100635 administered at a dose of 0.1 mg/kg had no effect. Co-administration of Lu AF21934 with WAY100635 resulted in the inhibition of Lu AF21934-induced behavioral phenotype in the social interaction test. Two-way ANOVA main effects analysis of the number of episodes revealed the significant effect of Lu AF21934 × WAY100635 interaction [F_(1.33)_ = 8.3, *P* < 0.006]. Post hoc Newman-Keuls analysis revealed the significant Lu AF21934 × WAY100635 interaction, *P* < 0.0004, when compared to Lu AF21934-treated group. Two-way ANOVA analysis of the time of interaction also revealed significant effect of Lu AF21934 × WAY100635 [F_(1.33)_ = 6.02; *P* < 0.01], and post hoc Newman-Keuls analysis revealed the significant effect of Lu AF21934 × WAY100635 interaction, *P* < 0.004, when compared to Lu AF21934-treated rats (Fig. 3a, b).

The control experiment with the groups of Lu AF21934, WAY100635, and Lu AF21934 + WAY100635 revealed that Lu AF21934, WAY100635, or Lu AF21934 + WAY100635 interaction had no influence on the number of episodes [F_(1.33)_ = 0.64] (Fig. 3a, b).

### The effect of combined administration of (*R*)-(+)-8-hydroxy-DPAT hydrobromide and a sub-effective dose of Lu AF21934 in the social interaction test in rats

Lu AF21934 was administered at a dose of 0.1 mg/kg, 60 min before the test and did not exhibit any effects. (*R*)-(+)-8-hydroxy-DPAT hydrobromide was given at a dose of 0.01 mg/kg, 15 min before the test, and also did not have any effects. Simultaneous administration of sub-effective doses of 5-HT_1A_ receptor agonist and mGlu_4_ receptor PAM-induced clear antipsychotic-like effects measured in two parameters. Two-way ANOVA main effect analysis of the number of episodes revealed the significant effect of Lu AF21934 × (*R*)-(+)-8-hydroxy-DPAT hydrobromide interaction [F_(1.32)_ = 7.7; *P* < 0.009]. Post hoc Newman-Keuls analysis revealed the significant effect of Lu AF21934 × (*R*)-(+)-8-hydroxy-DPAT hydrobromide only, *P* < 0.0001, when compared to all other groups. Two-way ANOVA analysis of the time of interaction revealed significant effect of Lu AF21934 × (*R*)-(+)-8-hydroxy-DPAT hydrobromide interaction [F_(1.32)_ = 20.08; *P* < 0.00008]. Post hoc Newman-Keuls analysis revealed the significant effect of Lu AF21934 × (*R*)-(+)-8-hydroxy-DPAT hydrobromide interaction only, *P* < 0.0001, when compared to all the other groups (Fig. 3c, d).

The control experiment with the groups of Lu AF21934, (*R*)-(+)-8-hydroxy-DPAT hydrobromide, and Lu AF21934 + (*R*)-(+)-8-hydroxy-DPAT hydrobromide revealed that neither Lu AF21934, (*R*)-(+)-8-hydroxy-DPAT hydrobromide, nor Lu AF21934 + (*R*)-(+)-8-hydroxy-DPAT hydrobromide interaction had any effect on the number of episodes [F_(1.33)_ = 0.76] (Fig. 3c, d).

### The effect of combined administration of lurasidone and a sub-effective dose of Lu AF21934 in the social interaction test in rats

**Fig. 4:**
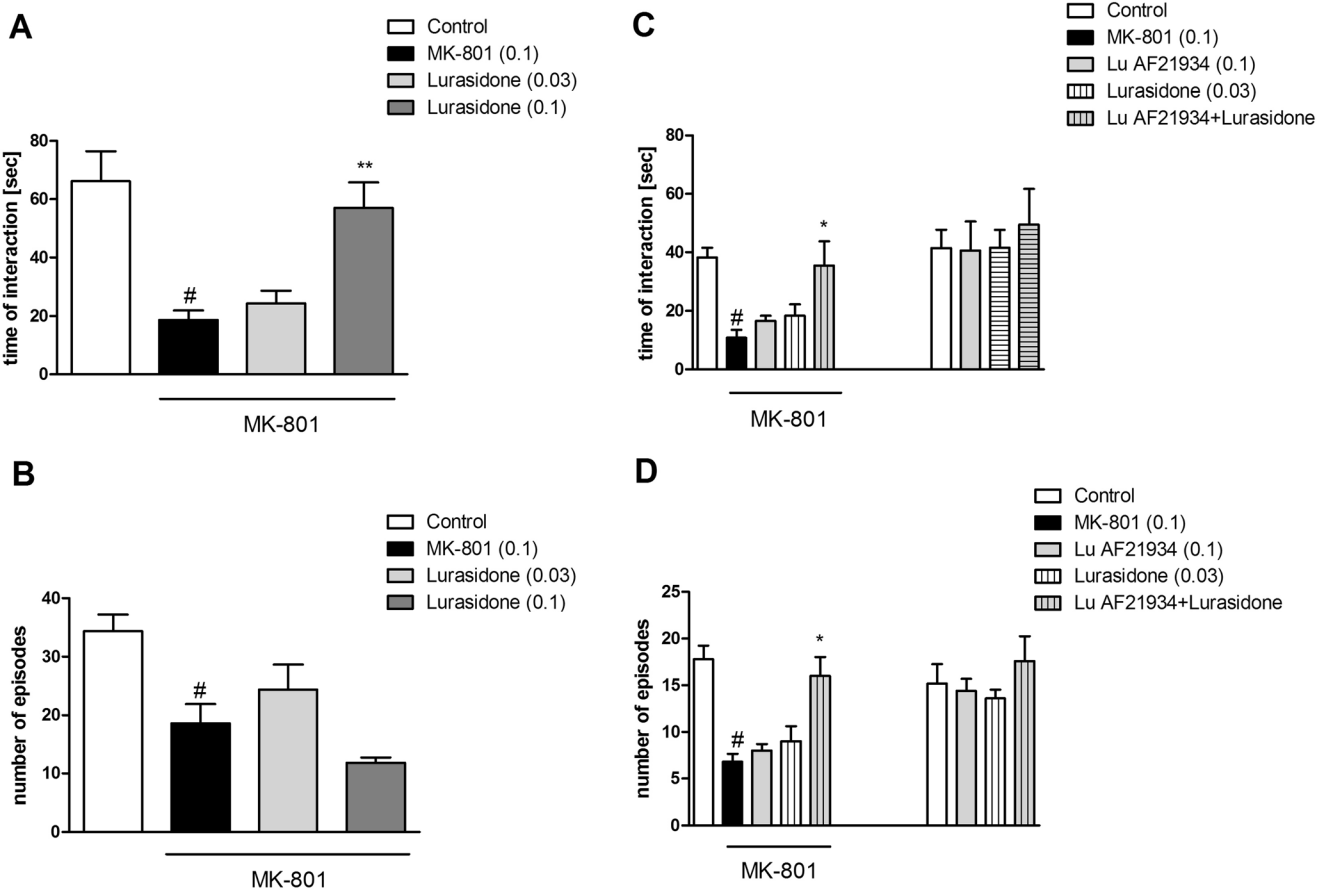
Effects on MK-801-induced deficits in social interaction. Dose-dependent effect of lurasidone alone (**a**, **b**) and the effect of combined administration of lurasidone and Lu AF21934 (**c**, **d**). Data are presented as means ± SEM. Doses in milligrams per kilogram are indicated in *parentheses*. #*P* < 0.05 versus controls, **P < 0.05 versus MK-801-treated group versus Lu AF21934-treated group

Lurasidone hydrochloride was administered at doses of 0.03 and 0.1 mg/kg. The drug administered at a dose of 0.1 mg/kg reversed MK-801-induced deficits only in the time of interaction parameter [F_(3.16)_ = 10.55; *P* < 0.0005] (Fig. 4a, b). Neither of the doses tested had any influence on the number of social episodes between rats.

The concomitant administration of ineffective doses of lurasidone (0.03 mg/kg) and Lu AF21934 (0.1 mg/kg) induced clear antipsychotic-like effect, increasing the duration of interactions between rats disrupted after MK-801 administration [F_(1.36)_ = 4.54; *P* < 0.03] and number of interactions [F_(1.36)_ = 9.41; *P* < 0.004] (Fig. 4c, d).

### The effect of combined administration of WAY100635 and Lu AF21934 in the novel object recognition test in rats

**Fig. 5:**
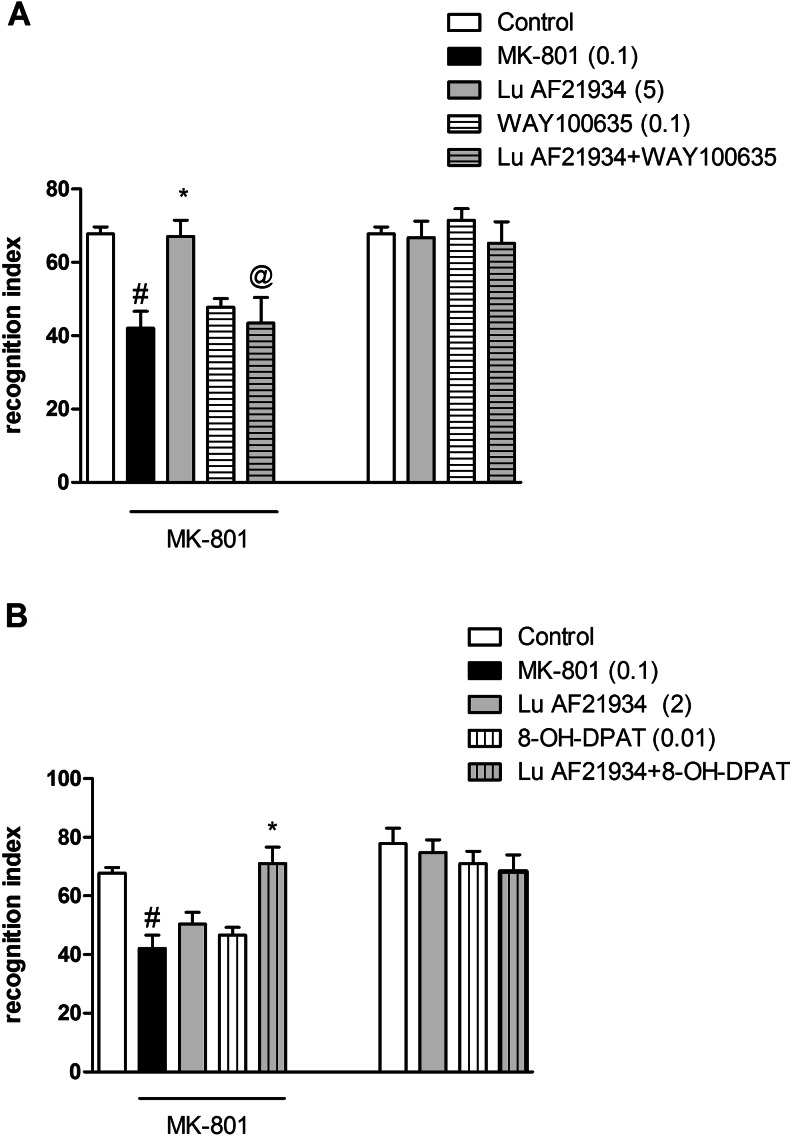
Effects on MK-801-induced deficits in NOR. The combined administration of Lu AF21934 (5 mg/kg) with WAY100635 (0.1 mg/kg) (**a**), and the co-administration of Lu AF21934 (0.5) with 8-OH-DPAT (0.01) (**b**) are presented. The control experiments with the same group treatments are also included. Data are presented as means ± SEM. Doses in milligrams per kilogram are indicated in *parentheses*. At least #*P* <0.05 versus controls, *P < 0.05 versus MK-801-treated group, @*P* < 0.05 versus Lu AF21934-treated group

Lu AF21934 was administered at a dose of 5 mg/kg, 60 min before the test, increasing the recognition index disturbed by MK-801 administration (*P* < 0.005). WAY100635 was given at a dose of 0.1 mg/kg, 45 min before the test, and did not have any effect. WAY100635, when dosed with Lu AF21934, antagonized Lu AF21934-induced behavioral phenotype in the NOR test. Two-way ANOVA main effects revealed statistical effect of Lu AF21934 × WAY100635 interaction [F_(1.28)_ = 9.17; *P* < 0.005]. Post hoc Newman-Keuls comparison revealed that WAY100635 antagonized Lu AF21934-induced effect, decreasing recognition index in a statistically significant way (*P* < 0.005), comparing to Lu AF21934-treated animals (Fig. 5a). The control experiment with the groups of Lu AF21934, WAY100635, and Lu AF21934 + WAY100635 revealed that neither of the combinations interaction had any influence on the recognition index [F_(1.28)_ = 0.92].

### The effect of combined administration of (*R*)-(+)-8-hydroxy-DPAT hydrobromide and a sub-effective dose of Lu AF21934 in the novel object recognition test in rats

Lu AF21934 was given at a dose of 2 mg/kg, 60 min before the test, and (*R*)-(+)-8-hydroxy-DPAT hydrobromide was given at a dose of 0.01 mg/kg, 15 min before the test. Neither drug had an effect when dosed alone. Concomitant administration of the sub-effective doses of Lu AF21934 and (*R*)-(+)-8-hydroxy-DPAT hydrobromide induced a clear antipsychotic-like phenotype in the NOR test. Two-way ANOVA main effects revealed statistical effect of Lu AF21934 × (*R*)-(+)-8-hydroxy-DPAT hydrobromide interaction [F_(1.28)_ = 7.4; *P* < 0.01]. Post hoc Newman-Keuls comparison revealed the statistical effect of Lu AF21934 × (*R*)-(+)-8-hydroxy-DPAT hydrobromide interaction, *P* < 0.00001 (Fig. 5b). The control experiment with the groups of Lu AF21934, (*R*)-(+)-8-hydroxy-DPAT hydrobromide and Lu AF21934 + (*R*)-(+)-8-hydroxy-DPAT hydrobromide revealed that neither of the combinations interaction had any influence on the recognition index [F_(1.28)_ = 0.65].

### The effect of combined administration of lurasidone hydrochloride and a sub-effective dose of Lu AF21934 in the novel object recognition test in rats

**Fig. 6:**
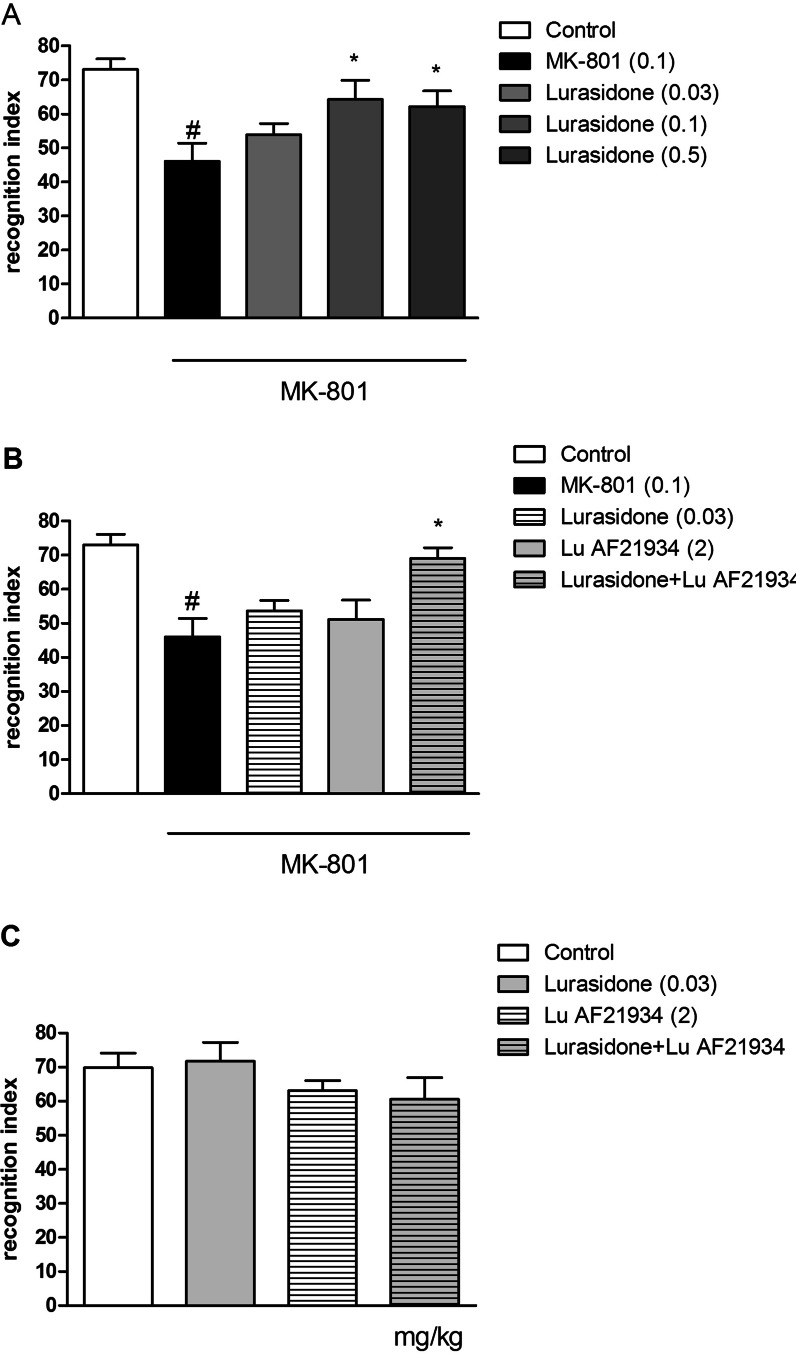
Effects on MK-801-induced deficits in NOR. The dose-dependent studies of lurasidone (**a**), and the co-administration of Lu AF21934 (2) with lurasidone (0.03) (**b**), as well as control experiments (**c**) are presented. Data are presented as means ± SEM. Doses in milligrams per kilogram are indicated in *parentheses*. At least #*P* < 0.05 versus controls, *P < 0.05 versus MK-801-treated group

Lurasidone was administered at doses of 0.03, 0.1, and 0.5 mg/kg (Fig. 6a). The lowest dose of 0.03 mg/kg was sub-effective, while two higher doses clearly induced the characteristic behavioral phenotype of the NOR paradigm [F_(4.43)_ = 4.89; *P* < 0.002]. Two-way ANOVA main effects revealed statistical effect of the concomitant administration of sub-effective doses of lurasidone (0.03 mg/kg) and Lu AF21934 (2 mg/kg), by increasing the recognition index that was disrupted by MK-801 administration [F_(1.35)_ = 4.9; *P* < 0.03]. Post hoc Newman-Keuls comparison revealed the statistical effect of Lu AF21934 × lurasidone interaction, *P* < 0.001 (Fig. 6b). None of the drug combinations had any effect when administered alone (Fig. 6c).

### The effect of combined administration of WAY100635 and Lu AF21934 in the spatial delayed alternation test in rats

**Fig. 7:**
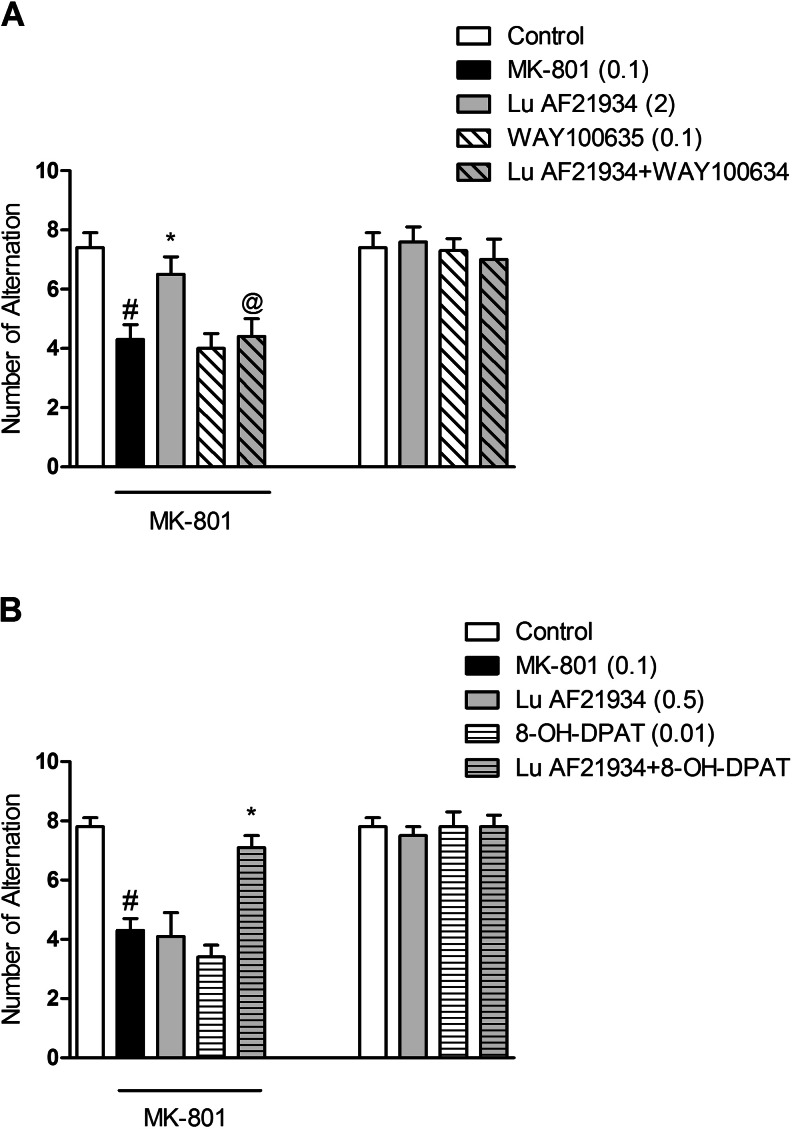
Effects on MK-801-induced deficits in SDA. The combined administration of Lu AF21934 (2 mg/kg) with WAY100635 (0.1 mg/kg) (**a**), and the co-administration of Lu AF21934 (0.5 mg/kg) with 8-OH-DPAT (0.01 mg/kg) (**b**) are presented. The control experiments are also included. Data are presented as means ± SEM. Doses in milligrams per kilogram are indicated in *parentheses*. At least #*P* < 0.05 versus controls, *P < 0.05 versus MK-801-treated group, @*P* < 0.05 versus Lu AF21934-treated group

Lu AF21934 was given at a dose of 5 mg/kg, 60 min before the test, inducing antipsychotic-like effects in the spatial delayed alteration (SDA) paradigm (*P* < 0.0007). WAY100635 was given at a dose of 0.1 mg/kg, 45 min before the test, having no own activity. WAY100635, when dosed with Lu AF21934, antagonized Lu AF21934-induced antipsychotic-like effect in the SDA test. Two-way ANOVA main effects revealed statistical effect of Lu AF21934 × WAY100635 interaction [F_(1.28)_ = 4.71; *P* < 0.03]. Post hoc Newman-Keuls comparison revealed that WAY100635 antagonized Lu AF21934-induced effect, *P* < 0.0007, comparing to Lu AF21934-treated animals (Fig. 7a). The control experiment with the groups of Lu AF21934, WAY100635, and Lu AF21934 + WAY100635 revealed that neither of the combinations had any effects on the behavior of the animals [F_(1.28)_ = 0.82].

### The effect of combined administration of (*R*)-(+)-8-hydroxy-DPAT hydrobromide and a sub-effective dose of Lu AF21934 in the spatial delayed alteration test in rats

Lu AF21934 was administered at a dose of 2 mg/kg, 60 min before the test, and did not induce any effects on its own. (*R*)-(+)-8-Hydroxy-DPAT hydrobromide was given at a dose of 0.01 mg/kg, 15 min before the test, and also had no effects. Concomitant administration of sub-effective doses of 5-HT_1A_ receptor agonist and the mGlu_4_ receptor PAM induced a clear SDA test phenotypic response. Two-way ANOVA main effects revealed statistical effect of Lu AF21934 × (*R*)-(+)-8-hydroxy-DPAT hydrobromide interaction [F_(1.28)_ = 13.32; *P* < 0.001]. Post hoc Newman-Keuls comparison revealed the statistical effect of Lu AF21934 × (*R*)-(+)-8-hydroxy-DPAT hydrobromide interaction, *P* < 0.001 (Fig. 7b). The control experiment with the groups of Lu AF21934, (*R*)-(+)-8-hydroxy-DPAT hydrobromide, and Lu AF21934 + (*R*)-(+)-8-hydroxy-DPAT hydrobromide revealed that none of the drug combinations had any influence on the rats behavior [F_(1.28)_ = 0.65].

## In vivo microdialysis

**Fig. 8:**
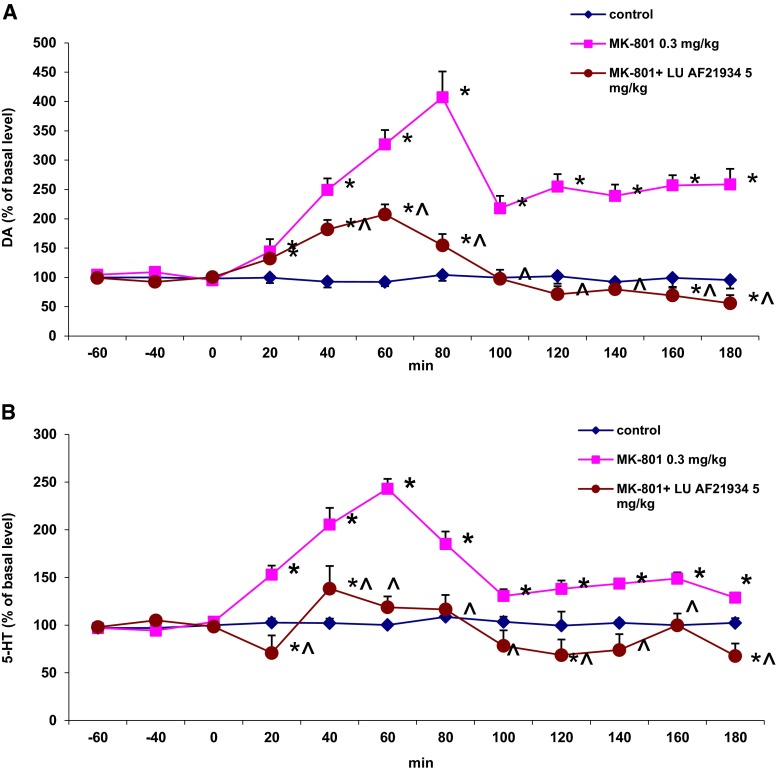
Effect of Lu AF21934 (5 mg/kg s.c) on extracellular concentration of dopamine (DA) (**a**) and 5-HT (**b**) in the rat prefrontal cortex. The figure shows time-course of the DA and 5-HT level between 20 and 180 min of the microdialysis sample collection. Values are presented as the mean ± SEM, *n* = 12–13 rats. *P < 0.05 and **P < 0.01 show significant differences from basal level

MK-801 at a dose of 0.3 mg/kg significantly increased DA and 5-HT in the rat frontal cortex reaching maximal effect at 80 and 60 min after administration, respectively (Fig. 8a, b). LU AF 21934 (5 mg/kg) attenuated increase in extracellular DA level induced by MK-801 (Fig. 8a). Repeated measures ANOVA showed significant effect of treatment [F_3,16_ = 196, *P* = 0], significant effect of time [F_8,128_ = 27, *P* = 0], and interaction between both factors [F_24,128_ = 15, *P* = 0].

Similarly, LU AF 21934 (5 mg/kg) lowered increased by MK-801 extracellular level of 5-HT (Fig. 8b). Repeated measures ANOVA showed significant effect of treatment [F_3,17_ = 54, *P* = 0], significant effect of time [F_8,136_ = 25, *P* = 0], and interaction between both factors [F_24,136_ = 8.2, *P* = 0].

Basal extracellular levels of DA and 5-HT (in pg/10 μl) in frontal cortex were, respectively, 1.50 ± 0.20 and 0.62 ± 0.06, and there were no significant differences observed between experimental groups.

## Discussion

The present studies expand on our recent reports showing that the selective mGlu_4_ receptor PAMs, Lu AF21934, and Lu AF32615, exhibited antipsychotic-like activity in animal models broadly used with drugs showing clinical efficacy in schizophrenia (Sławińska et al. 2013). Both of these compounds have been extensively characterized, and of special importance to this work, lack any cross-reactivity with 5-HT_1A_ receptors was reported (Bennouar et al. 2013). Herein, we elucidate aspects of the brain circuitry involved in the pharmacology on novel mGlu_4_ PAMs by exploiting the recently reported functional interactions between mGlu_4_ and 5-HT_1A_ receptors (Wierońska et al. 2013a, b). We used a number of animal models of schizophrenia, including MK-801-induced hyperactivity, DOI-induced head twitches, social interaction, novel object recognition, and spatial delayed alteration tests. In all of these procedures, the pharmacological cross-talk between mGlu_4_ and 5-HT_1A_ receptors was verified. Conceptually, these behavioral models may be considered as providing a phenotypic platform reporting on mGlu_4_ receptor activation in vivo by Lu AF21934.

The mechanism of action of all presently used neuroleptic drugs involves D_2_ receptor blockade, thought to play a critical role in reversing positive symptoms of psychosis (exaggerations and distortions of normal perception and thinking). However, D_2_ receptor blockade is not enough to reverse negative and cognitive psychotic symptoms, making typical neuroleptics such as haloperidol not efficient in these aspects. The novel antipsychotic drugs that tend to act on 5-HT_1A_ receptors, such as clozapine, olanzapine, risperidone, or lurasidone, demonstrated some benefits for negative symptoms in the clinic (Innamorati et al. 2013; Stauffer et al. 2012).

We recently reported our investigations on the role of 5-HT_1A_ receptors in the preclinical pharmacological actions of various mGlu receptor ligands (Wierońska et al. 2013a, b). The unique role of these receptors was proposed in preclinical models of cognitive disturbances of schizophrenia for atypical neuroleptics (APD) known to have agonist activity at the 5-HT_1A_ receptor (e.g., risperidon and lurasidone) (Nagai et al. 2009; Snigdha et al. 2011; Horiguchi and Meltzer 2013; Horiguchi et al. 2013). Such an activity is a common attribute with selective agonists at 5-HT_1A_ receptors, such as 8-OH-DPAT or tandospirone, which have shown efficacy in a number of animal models of schizophrenia, e.g., novel object recognition test (Bubeníková-Valesová et al. 2007; Horiguchi and Meltzer 2012; Horiguchi et al. 2012).

In our present investigations, we used a number of animal models thought to represent positive, negative, and cognitive symptoms of schizophrenia. As previously, we used MK-801-induced hyperactivity, DOI-induced head twitches, the social interaction test, novel object recognition test, and spatial delayed alteration task. This wide range of methods may reflect on the main aspects of the pathophysiology of schizophrenia, mirroring the clinical picture of the disease, and allowing for broadly conceived behavioral research. Historically, these standard pharmacological protocols have worked well in our laboratories, they all are well-known and well-established procedures with excellent predictive validity, e.g., positive response is observed predominantly for antipsychotic drugs, and not for other class of psychotropics, such as antidepressants or anxiolytics.

In our studies, a number of well-known, validated chemical probes were used, such as WAY100635 as a selective inhibitor of the 5-HT_1A_ receptor-mediated signaling, and the agonist (*R*)-(+)-8-hydroxy-DPAT as a stimulator of the signaling (Foster and Goa 1999; Cosi and Koek 2000). We also used lurasidone, an antipsychotic drug recently receiving FDA approval, that has strong affinity toward 5-HT_1A_ receptors (*K*_i_ = 6.8 nM) and demonstrated partial agonist functionality (Horiguchi and Meltzer 2012; Horiguchi et al. 2012).

Previously, we showed that low doses of 5-HT_1A_ receptor ligands inhibited (WAY100635) or enhanced ((*R*)-8-hydroxy-DPAT) the pharmacological effects of LSP1-2111, a brain penetrant, mGlu_4_-preferred orthosteric agonist (Wierońska et al. 2013a, b; Cajina et al. 2014). We used the 5-HT_1A_ receptor antagonist WAY100635 at a dose of 0.1 mg/kg, and the agonist (*R*)-(+)-8-hydroxy-DPAT at a dose of 0.01 mg/kg. At that dose, WAY100635 inhibited the action of an effective dose of LSP1-2111, while a sub-effective dose of (*R*)-(+)-8-hydroxy-DPAT given together with a sub-effective dose of LSP1-2111, enhanced its action (Wierońska et al. 2013a, b). Higher doses of WAY100635 (1 mg/kg and above) or (*R*)-(+)-8-hydroxy-DPAT (0.5 mg/kg and above) induced their own effects, namely, antipsychotic-like behaviors or the so-called serotonergic syndrome, respectively. It is worth to mention that (*R*)-(+)-8-hydroxy-DPAT given at the dose of 0.025 mg/kg had clear antipsychotic-like action.

In our present studies, the actions of Lu AF21934 were inhibited by WAY100635 in all tests conducted, suggesting that in these models, 5-HT_1A_ receptors play an important mechanistic role in the expression of Lu AF21934-mediated effects, independently of the mechanism of mGlu_4_ receptor activation.

To further establish the role of the 5-HT_1A_ receptor in the actions mediated by Lu AF21934, a sub-effective dose of (*R*)-(+)-8-hydroxy-DPAT, together with a sub-effective dose of Lu AF21934, were administered. As seen previously with LSP1-2111, the drugs combination reversed MK-801-induced dysfunctions, decreasing the hyperactivity and increasing social behavior in the social interaction test. The improvement of the recognition index in the NOR paradigm and the reversal of the SDA dysfunction were also evident. The drugs combinations had no any own effects when tested in separated tests.

In this work, we present mechanistic evidence of an interaction between the 5-HT_1A_ and mGlu_4_ receptors, and we propose a multidirectional action of the novel class of neuroleptics. Marketed antipsychotics are multimodal drugs, targeting several receptors. For example, lurasidone is an antipsychotic recently approved in the USA and Canada for the treatment of schizophrenia and bipolar depression. Like other atypical antipsychotics, such as ziprasidone and clozapine, lurasidone is a high-affinity partial agonist at the 5-HT_1A_ receptor with known efficacy in preclinical studies in some animal models of psychotic disturbances (Schmidt et al. 2001; Sprouse et al. 1999; Horiguchi and Meltzer 2012; Horiguchi et al. 2012). To gain support for our developing hypothesis of mGlu_4_ receptor/5-HT_1A_ receptor interactions, we tested the novel antipsychotic lurasidone in preclinical models thought to represent the positive, negative and cognitive disturbances (DOI-induced head twitches, social interaction and novel object recognition test, respectively). While lurasidone has been widely discussed in the field for its efficacy in a model of cognitive disturbances (novel object recognition test, Horiguchi et al. 2012), to our best knowledge, no reports exist on its effectiveness in animal models thought to reflect positive and negative symptoms. Therefore, this work for the first time shows the efficacy of lurasidone in DOI-induced head twitches and in the social interaction models. However, in the latter procedure, the drug reversed MK-801-induced deficit in time of interaction and not in the number of episodes. The compound was active in a relatively narrow range of doses. In the combination studies, we used lurasidone at the sub-effective dose of 0.03 mg/kg, together with a sub-effective dose of Lu AF21934, relevant to each particular test. In all of these procedures, we showed that the combination effectively reversed all phenotypes induced by psychotomimetic agents. Interestingly, in the social interaction test, the combination was effective also in reversing deficit in the number of episodes, not influenced by lurasidone alone. All those results suggest a crosstalk between mGlu_4_ and 5-HT_1A_ receptors, with potential implications for antipsychotic medications.

We are aware that the pharmacological tools used in the present paper are not enough to claim whether 5-HT_1A_ receptors are necessary for mGlu_4_-based expression of the phenotypic responses explored herein. However, the results constitute strong support for our previous studies with orthosteric, preferential mGlu_4_ agonists, LSP1-2111, suggesting the possibility of the existence of a pharmacological crosstalk between mGlu_4_ and 5-HT_1A_ receptors.

Considering the putative mechanism of this interaction, the theory of antipsychotic activity of mGlu ligands raised by Conn et al. (2009) and the data on the distribution of 5-HT_1A_ receptors in the brain will be analyzed. According to Conn’s theory, schizophrenia is a consequence of the dysfunction of NMDA receptors expressed postsynaptically on GABAergic interneurons. The following loss of GABAergic inhibition over glutamatergic thalamocortical projections results in the enhancement of glutamate release. Presynaptically expressed mGlu_4_ receptors would counteract this increased glutamatergic activity (see: Conn et al. 2009; Sławińska et al. 2013).

On the other hand, increased level of serotonin is also thought to be responsible for the induction of psychotic symptoms. Variety of studies, including our present results, show that MK-801 may induce increase not only in dopamine but also serotonin release that presumably contributes to the observed MK-801-induced disturbations (López-Gil et al. 2009; Wedzony et al. 1993). With the use of in vivo microdialysis studies, we showed that MK-801-induced increase in dopamine and, most importantly, serotonin release in the prefrontal cortex was counteracted by the Lu AF21934 administration, supporting the hypothesis of the interaction between serotonergic system and mGlu_4_ receptors in MK-801 evoked actions.

The exact mechanism, by which mGlu_4_ receptors activation contributes to inhibition of that serotonergic outflow from terminals projecting to the cortex, and the role of 5-HT_1A_ receptors in that chain of events remain to be explored. However, the existence of such an interaction may have important practical connotations, as the use of two different compounds exerting therapeutic efficacy in combination creates novel treatment opportunities, which may be more effective and less burdened with the risk of adverse effects than monotherapy.

In summary, we propose that mGlu_4_ and 5-HT_1A_ receptors play synergistically in the regulation of glutamatergic activity in the CNS and therefore may constitute a dual target for putative antipsychotic treatment.

## Electronic supplementary material

Below is the link to the electronic supplementary material.

ESM 1 (DOCX 44 kb)
