# Supplementary material for: The antipsychotic-like effects in rodents of the positive allosteric modulator Lu AF21934 involve 5-HT1A receptor signaling: mechanistic studies
Source: Psychopharmacology (Berl). 2014 Jul 11;232(1):259–73. doi: 10.1007/s00213-014-3657-4 (PMC4281359; doi:10.1007/s00213-014-3657-4)
Supplement: Supplementary file 1 — (DOCX 44 kb) [file 213_2014_3657_JobSheetMOESM1_ESM.docx]

Suplementary materials:

STATISTICAL ANALYSIS OF THE RESULTS

Fig. 1 MK-801 induced hyperactivity (ambulation scores)

Two-way ANOVA analysis (STATISTICA):

Lu AF21934+WAY100635

Main effects:

| Effects | Univariate Tests of Significance for Var1  Sigma-restricted parametrization  Effective hypothesis decomposition | | | | |
| --- | --- | --- | --- | --- | --- |
|  | SS | Degr. Of Freedom | MS | F | p |
| Intercept | 1,326956E+09 | 1 | 1,326956E+09 | 148,5470 | 0,000000 |
| LuAF21934 | 1,096247E+07 | 1 | 1,096247E+07 | 1,2272 | 0,277056 |
| WAY100635 | 9,208460E+07 | 1 | 9,208460E+07 | 10,3085 | 0,003229 |
| LuAF21934+WAY100635 | 7,167692E+07 | 1 | 7,167692E+07 | 8,0239 | 0,008311 |
| Error | 2,590540E+08 | 29 | 8,932898E+06 |  |  |

Post-hoc analysis

| Cell No. | Newman-Keuls test; variable Var 1  Approximate Probabilities for Post Hoc Tests  Error:Between MS=8933E3, df=29,000 | | | | | |
| --- | --- | --- | --- | --- | --- | --- |
|  | LuAF21934 | WAY100635 | 1  6729,6 | 2  7123,5 | 3  2624,0 | 4  8920,7 |
| 1 | 0 | 0 |  | 0,791240 | 0,009446 | 0,311867 |
| 2 | 0 | 1 | 0,791240 |  | 0,013041 | 0,232539 |
| 3 | 1 | 0 | 0,009446 | 0,013041 |  | 0,001151 |
| 4 | 1 | 1 | 0,311867 | 0,232539 | 0,001151 |  |

Lu AF21934+ RS-8-OH-DPAT

Main effects:

| Effects | Univariate Tests of Significance for Var1  Sigma-restricted parametrization  Effective hypothesis decomposition | | | | |
| --- | --- | --- | --- | --- | --- |
|  | SS | Degr. Of Freedom | MS | F | p |
| Intercept | 2,491702E+09 | 1 | 2,491702E+09 | 162,7827 | 0,000000 |
| LuAF21934 | 1,136540E+08 | 1 | 1,136540E+08 | 7,4250 | 0,010787 |
| RS-8-OH-DPAT | 1,974276E+07 | 1 | 1,974276E+07 | 1,2898 | 0,265385 |
| LuAF21934+ RS-8-OH-DPAT | 7,825148E+07 | 1 | 7,825148E+07 | 5,1122 | 0,031436 |
| Error | 4,439007E+08 | 29 | 1,530692E+07 |  |  |

Post-hoc analysis

| Cell No. | Newman-Keuls test; variable Var 1  Approximate Probabilities for Post Hoc Tests  Error:Between MS=1531E4, df=29,000 | | | | | |
| --- | --- | --- | --- | --- | --- | --- |
|  | LuAF21934 | RS-8-OH-DPAT | 1  9791,6 | 2  11326 | 3  9158,9 | 4  4526,1 |
| 1 | 0 | 0 |  | 0,432810 | 0,745383 | 0,028028 |
| 2 | 0 | 1 | 0,432810 |  | 0,507592 | 0,007462 |
| 3 | 1 | 0 | 0,745383 | 0,507592 |  | 0,023071 |
| 4 | 1 | 1 | 0,028028 | 0,007462 | 0,023071 |  |

Fig. 2 DOI-induced head twitches

Lu AF21934+ WAY100635

Main effects:

| Effects | Univariate Tests of Significance for Var1  Sigma-restricted parametrization  Effective hypothesis decomposition | | | | |
| --- | --- | --- | --- | --- | --- |
|  | SS | Degr. Of Freedom | MS | F | p |
| Intercept | 3735,202 | 1 | 3735,202 | 253,1070 | 0,000000 |
| LuAF21934 | 16,126 | 1 | 16,126 | 1,0927 | 0,305491 |
| WAY100635 | 28,601 | 1 | 28,601 | 1,9381 | 0,175673 |
| LuAF21934+ WAY100635 | 139,619 | 1 | 139,619 | 9,4610 | 0,004892 |
| Error | 383,692 | 26 | 14,757 |  |  |

Post-hoc analysis

| Cell No. | Newman-Keuls test; variable Var 1  Approximate Probabilities for Post Hoc Tests  Error:Between MS=14,757, df=26,000 | | | | | |
| --- | --- | --- | --- | --- | --- | --- |
|  | LuAF21934 | WAY100635 | 1  13,222 | 2  10,833 | 3  7,3750 | 4  13,714 |
| 1 | 0 | 0 |  | 0,244700 | 0,019262 | 0,808322 |
| 2 | 0 | 1 | 0,244700 |  | 0,096798 | 0,337936 |
| 3 | 1 | 0 | 0,019262 | 0,096798 |  | 0,019623 |
| 4 | 1 | 1 | 0,808322 | 0,337936 | 0,019623 |  |

Lu AF21934+ RS-8-OH-DPAT

Main effects:

| Effects | Univariate Tests of Significance for Var1  Sigma-restricted parametrization  Effective hypothesis decomposition | | | | |
| --- | --- | --- | --- | --- | --- |
|  | SS | Degr. Of Freedom | MS | F | p |
| Intercept | 3997,496 | 1 | 3997,496 | 259,7398 | 0,000000 |
| LuAF21934 | 61,334 | 1 | 61,334 | 3,9852 | 0,056474 |
| RS-8-OH-DPAT | 259,496 | 1 | 259,496 | 16,8609 | 0,000354 |
| LuAF21934+ RS-8-OH-DPAT | 127,009 | 1 | 127,009 | 8,2525 | 0,007998 |
| Error | 400,150 | 26 | 15,390 |  |  |

Post-hoc analysis

| Cell No. | Newman-Keuls test; variable Var 1  Approximate Probabilities for Post Hoc Tests  Error:Between MS=15,390, df=26,000 | | | | | |
| --- | --- | --- | --- | --- | --- | --- |
|  | LuAF21934 | RS-8-OH-DPAT | 1  14,9 | 2  13,00 | 3  16,25 | 4  5,5 |
| 1 | 0 | 0 |  | 0,391250 | 0,540969 | 0,000687 |
| 2 | 0 | 1 | 0,391250 |  | 0,311182 | 0,002102 |
| 3 | 1 | 0 | 0,540969 | 0,311182 |  | 0,000364 |
| 4 | 1 | 1 | 0,000687 | 0,002102 | 0,000364 |  |

Lurasidone: dose-dependence

One-way analysis of variance

P value: 0.0002

P value summary: ***

Number of groups: 3

F: 13.45

R square: 0.5861

Bartlett’s test for equal variances:

Bartlett’s statistic (corrected): 5.135

P value: 0.0767

P value summary: ns

ANOVA table

|  | SS | DF | MS |
| --- | --- | --- | --- |
| Treatment (between columns) | 293,8 | 2 | 146,9 |
| Residula (within columns) | 207,5 | 19 | 10,92 |
| Total | 501,3 | 21 |  |
|  |  |  |  |
| Dunnett’s Multiple Comparison Test | Mean Diff | q | summary |
| Control vs Lurasidone 0.03 | -0,7500 | 0,4202 | Ns |
| Control vs Lurasidone 0.1 | 7,250 | 4,388 | *** |

Lurasidone + Lu AF21934

Main effects:

| Effects | Univariate Tests of Significance for Var1  Sigma-restricted parametrization  Effective hypothesis decomposition | | | | |
| --- | --- | --- | --- | --- | --- |
|  | SS | Degr. Of Freedom | MS | F | p |
| Intercept | 4203,247 | 1 | 4203,247 | 600,9181 | 0,000000 |
| LuAF21934 | 176,436 | 1 | 176,436 | 25,2242 | 0,000029 |
| Lurasidone | 87,006 | 1 | 87,006 | 12,4388 | 0,001524 |
| LuAF21934+ Lurasidone | 51,156 | 1 | 51,156 | 7,3135 | 0,011701 |
| Error | 188,857 | 27 | 6,995 |  |  |

Post-hoc analysis

| Cell No. | Newman-Keuls test; variable Var 1  Approximate Probabilities for Post Hoc Tests  Error:Between MS=6,9947, df=27,000 | | | | | |
| --- | --- | --- | --- | --- | --- | --- |
|  | LuAF21934 | Lurasidone | 1  14,9 | 2  13,00 | 3  16,25 | 4  5,5 |
| 1 | 0 | 0 |  | 0,565782 | 0,247102 | 0,0,000173 |
| 2 | 0 | 1 | 0,0,565782 |  | 0,0,299798 | 0,0,000147 |
| 3 | 1 | 0 | 0,0,247102 | 0,299798 |  | 0,000282 |
| 4 | 1 | 1 | 0,000173 | 0,000147 | 0,000282 |  |

Fig. 3 Social interaction:

Lu AF21934+WAY100635

Number of episodes:

Main effects:

| Effects | Univariate Tests of Significance for Var1  Sigma-restricted parametrization  Effective hypothesis decomposition | | | | |
| --- | --- | --- | --- | --- | --- |
|  | SS | Degr. Of Freedom | MS | F | p |
| Intercept | 1478,054 | 1 | 1478,054 | 251,8254 | 0,000000 |
| LuAF21934 | 118,502 | 1 | 118,502 | 20,19 | 0,000081 |
| WAY100635 | 44,617 | 1 | 44,617 | 7,6017 | 0,009428 |
| LuAF21934+ WAY100635 | 48,754 | 1 | 48,754 | 8,3066 | 0,006897 |
| Error | 193,689 | 33 | 5,869 |  |  |

Post-hoc analysis

| Cell No. | Newman-Keuls test; variable Var 1  Approximate Probabilities for Post Hoc Tests  Error:Between MS=5,8694, df=33,000 | | | | | |
| --- | --- | --- | --- | --- | --- | --- |
|  | LuAF21934 | WAY100635 | 1  4,5 | 2  4,6 | 3  10,4 | 4  5,8889 |
| 1 | 0 | 0 |  | 0,930195 | 0,000208 | 0,445721 |
| 2 | 0 | 1 | 0,930195 |  | 0,000157 | 0,262902 |
| 3 | 1 | 0 | 0,000208 | 0,000157 |  | 0,000457 |
| 4 | 1 | 1 | 0,445721 | 0,262902 | 0,000457 |  |

Lu AF21934+WAY100635

Time of interaction:

Main effects:

| Effects | Univariate Tests of Significance for Var1  Sigma-restricted parametrization  Effective hypothesis decomposition | | | | |
| --- | --- | --- | --- | --- | --- |
|  | SS | Degr. Of Freedom | MS | F | p |
| Intercept | 10106,04 | 1 | 10106,04 | 116,0719 | 0,000000 |
| LuAF21934 | 742,00 | 1 | 742,00 | 8,5222 | 0,006185 |
| WAY100635 | 324,21 | 1 | 324,21 | 3,7237 | 0,062023 |
| LuAF21934+ WAY100635 | 524,25 | 1 | 524,25 | 6,0212 | 0,019414 |
| Error | 2960,28 | 33 | 87,07 |  |  |

Post-hoc analysis

| Cell No. | Newman-Keuls test; variable Var 1  Approximate Probabilities for Post Hoc Tests  Error:Between MS=87,067, df=33,000 | | | | | |
| --- | --- | --- | --- | --- | --- | --- |
|  | LuAF21934 | WAY100635 | 1  13,222 | 2  10,833 | 3  7,3750 | 4  13,714 |
| 1 | 0 | 0 |  | 0,713359 | 0,003185 | 0,765353 |
| 2 | 0 | 1 | 0,713359 |  | 0,004557 | 0,744196 |
| 3 | 1 | 0 | 0,003185 | 0,004557 |  | 0,004025 |
| 4 | 1 | 1 | 0,765353 | 0,744196 | 0,004025 |  |

Lu AF21934+RS-8-OH-DPAT

Number of episodes:

Main effects:

| Effects | Univariate Tests of Significance for Var1  Sigma-restricted parametrization  Effective hypothesis decomposition | | | | |
| --- | --- | --- | --- | --- | --- |
|  | SS | Degr. Of Freedom | MS | F | p |
| Intercept | 1019,145 | 1 | 1019,145 | 153,1418 | 0,000000 |
| LuAF21934 | 133,201 | 1 | 133,201 | 20,0155 | 0,000091 |
| RS-8-OH-DPAT | 137,145 | 1 | 137,145 | 20,6081 | 0,000075 |
| LuAF21934+ RS-8-OH-DPAT | 51,257 | 1 | 51,257 | 7,7022 | 0,009134 |
| Error | 212,957 | 32 | 6,655 |  |  |

Post-hoc analysis

| Cell No. | Newman-Keuls test; variable Var 1  Approximate Probabilities for Post Hoc Tests  Error:Between MS=6,6549, df=32,000 | | | | | |
| --- | --- | --- | --- | --- | --- | --- |
|  | LuAF21934 | RS-8-OH-DPAT | 1  2,6667 | 2  4,2 | 3  4,1429 | 4  10,500 |
| 1 | 0 | 0 |  | 0,434712 | 0,238647 | 0,000166 |
| 2 | 0 | 1 | 0,434712 |  | 0,963317 | 0,000145 |
| 3 | 1 | 0 | 0,238647 | 0,963317 |  | 0,000151 |
| 4 | 1 | 1 | 0,000166 | 0,000145 | 0,000151 |  |

Lu AF21934+RS-8-OH-DPAT

Time of interaction:

Lu AF21934+ RS-8-OH-DPAT

Main effects:

| Effects | Univariate Tests of Significance for Var1  Sigma-restricted parametrization  Effective hypothesis decomposition | | | | |
| --- | --- | --- | --- | --- | --- |
|  | SS | Degr. Of Freedom | MS | F | p |
| Intercept | 6913,204 | 1 | 6913,204 | 205,7177 | 0,000000 |
| LuAF21934 | 604,873 | 1 | 604,873 | 17,9993 | 0,000168 |
| RS-8-OH-DPAT | 481,548 | 1 | 481,548 | 14,3295 | 0,000616 |
| LuAF21934+ RS-8-OH-DPAT | 675,077 | 1 | 675,077 | 20,0884 | 0,000084 |
| Error | 1108,975 | 32 | 33,605 |  |  |

Post-hoc analysis

| Cell No. | Newman-Keuls test; variable Var 1  Approximate Probabilities for Post Hoc Tests  Error:Between MS=33,605, df=32,000 | | | | | |
| --- | --- | --- | --- | --- | --- | --- |
|  | LuAF21934 | RS-8-OH-DPAT | 1  10,33 | 2  9,00 | 3  9,87 | 4  25,7 |
| 1 | 0 | 0 |  | 0,875404 | 0,866691 | 0,000124 |
| 2 | 0 | 1 | 0,875404 |  | 0,748690 | 0,000165 |
| 3 | 1 | 0 | 0,866691 | 00,748690 |  | 0,000129 |
| 4 | 1 | 1 | 0,000124 | 0,000165 | 0,000129 |  |

Fig. 4 Social interaction:

Lurasidone: dose-dependence-number of episodes

One-way analysis of variance

P value: 0.0007

P value summary: ***

Number of groups: 4

F: 9,738

R square: 0.6461

Bartlett’s test for equal variances:

Bartlett’s statistic (corrected): 6,556

P value: 0.0875

P value summary: ns

ANOVA table

|  | SS | DF | MS |
| --- | --- | --- | --- |
| Treatment (between columns) | 1374 | 3 | 457,9 |
| Residula (within columns) | 752,4 | 16 | 47,03 |
| Total | 2126 | 19 |  |
|  |  |  |  |
| Newman-Keuls Multiple Comparison Test | Mean Diff | q | summary |
| Lurasidone (0.1) vs Control | -22,60 | 7,369 | *** |
| Lurasidone (0.1) vs Lurasidone (0.03) | -12,60 | 4,109 | * |
| Lurasidone (0.1) vs MK-801 (0.1) | -6,800 | 2,217 | ns |
| MK-801 (0.1) vs Control | -15,80 | 5,152 | ** |
| MK-801 (0.1) vs Lurasidone (0.03) | -5,800 | 1,891 | ns |
| Lurasidone (0.03) vs Control | -10,00 | 3,261 | * |

Lurasidone: dose-dependence-time of interaction

One-way analysis of variance

P value: 0.0005

P value summary: ***

Number of groups: 4

F: 10,55

R square: 0.6642

Bartlett’s test for equal variances:

Bartlett’s statistic (corrected): 5,728

P value: ns

P value summary: ns

ANOVA table

|  | SS | DF | MS |
| --- | --- | --- | --- |
| Treatment (between columns) | 8336 | 3 | 2779 |
| Residula (within columns) | 4215 | 16 | 263,5 |
| Total | 12551 | 19 |  |
|  |  |  |  |
| Newman-Keuls Multiple Comparison Test | Mean Diff | q | summary |
| MK-801 (0.1) vs Control | -47,60 | 6,558 | ** |
| MK-801 (0.1) vs Lurasidone (0.1) | -38,40 | 5,290 | ** |
| MK-801 (0.1) vs Lurasidone (0.03) | -5,800 | 0,7990 | ns |
| Lurasidone (0.03) vs Control | -41,80 | 5,759 | ** |
| Lurasidone (0.03) vs Lurasidone (0.1) | -32,60 | 4,491 | ** |
| Lurasidone (0.1) vs Control | -9,200 | 1,267 | ns |

Lu AF21934+Lurasidone

Number of episodes:

Main effects:

| Effects | Univariate Tests of Significance for Var1  Sigma-restricted parametrization  Effective hypothesis decomposition | | | | |
| --- | --- | --- | --- | --- | --- |
|  | SS | Degr. Of Freedom | MS | F | p |
| Intercept | 3960,100 | 1 | 3960,100 | 443,2948 | 0,000000 |
| LuAF21934 | 168,100 | 1 | 168,100 | 18,8172 | 0,000111 |
| Lurasidone | 260,100 | 1 | 260,100 | 29,1157 | 0,000004 |
| LuAF21934+ Lurasidone | 84,100 | 1 | 84,100 | 9,4142 | 0,004075 |
| Error | 321,6 | 36 | 8,933 |  |  |

Post-hoc analysis

| Cell No. | Newman-Keuls test; variable Var 1  Approximate Probabilities for Post Hoc Tests  Error:Between MS=8,9333, df=36,000 | | | | | |
| --- | --- | --- | --- | --- | --- | --- |
|  | LuAF21934 | Lurasidone | 1  6,8 | 2  9,0 | 3  8,0 | 4  16,0 |
| 1 | 0 | 0 |  | 0,240036 | 0,375415 | 0,000159 |
| 2 | 0 | 1 | 0,240036 |  | 0,459377 | 0,000127 |
| 3 | 1 | 0 | 0,375415 | 0,459377 |  | 0,000128 |
| 4 | 1 | 1 | 0,000159 | 0,000127 | 0,000128 |  |

Lu AF21934+ Lurasidone

Time of interaction:

Main effects:

| Effects | Univariate Tests of Significance for Var1  Sigma-restricted parametrization  Effective hypothesis decomposition | | | | |
| --- | --- | --- | --- | --- | --- |
|  | SS | Degr. Of Freedom | MS | F | p |
| Intercept | 15681,6 | 1 | 15681,6 | 163,5014 | 0,000000 |
| LuAF21934 | 1537,6 | 1 | 1537,6 | 16,0315 | 0,000298 |
| Lurasidone | 1488,4 | 1 | 1488,4 | 15,5185 | 0,000360 |
| LuAF21934+ Lurasidone | 435,6 | 1 | 435,6 | 4,5417 | 0,039977 |
| Error | 3452,8 | 36 | 95,91 |  |  |

Post-hoc analysis

| Cell No. | Newman-Keuls test; variable Var 1  Approximate Probabilities for Post Hoc Tests  Error:Between MS=33,605, df=32,000 | | | | | |
| --- | --- | --- | --- | --- | --- | --- |
|  | LuAF21934 | Lurasidone | 1  10,33 | 2  9,00 | 3  9,87 | 4  25,7 |
| 1 | 0 | 0 |  | 0,209318 | 0,391318 | 0,000173 |
| 2 | 0 | 1 | 0,209318 |  | 0,963943 | 0,000427 |
| 3 | 1 | 0 | 0,391318 | 0,963943 |  | 0,000240 |
| 4 | 1 | 1 | 0,000173 | 0,000427 | 0,000240 |  |

Fig. 5 Novel object recognition test

Lu AF21934+ WAY100635

Main effects:

| Effects | Univariate Tests of Significance for Var1  Sigma-restricted parametrization  Effective hypothesis decomposition | | | | |
| --- | --- | --- | --- | --- | --- |
|  | SS | Degr. Of Freedom | MS | F | p |
| Intercept | 80400,5 | 1 | 80400,5 | 431,3290 | 0,000000 |
| LuAF21934 | 861,13 | 1 | 861,13 | 4,6197 | 0,040394 |
| WAY100635 | 648 | 1 | 648 | 3,4764 | 0,072766 |
| LuAF21934+ WAY100635 | 1711,13 | 1 | 1711,13 | 9,1798 | 0,005217 |
| Error | 5219,25 | 28 | 186,4 |  |  |

Post-hoc analysis

| Cell No. | Newman-Keuls test; variable Var 1  Approximate Probabilities for Post Hoc Tests  Error:Between MS=186,4, df=28,000 | | | | | |
| --- | --- | --- | --- | --- | --- | --- |
|  | LuAF21934 | WAY100635 | 1  42,125 | 2  47,75 | 3  67,125 | 4  43,5 |
| 1 | 0 | 0 |  | 0,691585 | 0,005470 | 0,841948 |
| 2 | 0 | 1 | 0,691585 |  | 0,008496 | 0,538734 |
| 3 | 1 | 0 | 0,005470 | 0,008496 |  | 0,004905 |
| 4 | 1 | 1 | 0,841948 | 0,538734 | 0,004905 |  |

Lu AF21934+ RS-8-OH-DPAT

Main effects:

| Effects | Univariate Tests of Significance for Var1  Sigma-restricted parametrization  Effective hypothesis decomposition | | | | |
| --- | --- | --- | --- | --- | --- |
|  | SS | Degr. Of Freedom | MS | F | p |
| Intercept | 85078,13 | 1 | 85078,13 | 878,3068 | 0,000000 |
| LuAF21934 | 2775,12 | 1 | 2775,12 | 28,6491 | 0,000011 |
| RS-8-OH-DPAT | 1860,5 | 1 | 1860,5 | 19,2069 | 0,000150 |
| LuAF21934+ RS-8-OH-DPAT | 722,0 | 1 | 722,0 | 7,4536 | 0,010824 |
| Error | 2712,25 | 28 | 96,87 |  |  |

Post-hoc analysis

| Cell No. | Newman-Keuls test; variable Var 1  Approximate Probabilities for Post Hoc Tests  Error:Between MS=15,390, df=26,000 | | | | | |
| --- | --- | --- | --- | --- | --- | --- |
|  | LuAF21934 | RS-8-OH-DPAT | 1  39,375 | 2  45,125 | 3  48,500 | 4  73,25 |
| 1 | 0 | 0 |  | 0,252591 | 0,170962 | 0,000165 |
| 2 | 0 | 1 | 0,252591 |  | 0,498600 | 0,000133 |
| 3 | 1 | 0 | 0,170962 | 0,498600 |  | 0,000163 |
| 4 | 1 | 1 | 0,000165 | 0,000133 | 0,000163 |  |

Fig. 6 Novel object recognition:

Lurasidone: dose-dependence-

One-way analysis of variance

P value: 0.0024

P value summary: **

Number of groups: 5

F: 4,891

R square: 0,3127

Bartlett’s test for equal variances:

Bartlett’s statistic (corrected): 5,428

P value: 0.2461

P value summary: ns

ANOVA table

|  | SS | DF | MS |
| --- | --- | --- | --- |
| Treatment (between columns) | 4041 | 4 | 1010 |
| Residula (within columns) | 8882 | 43 | 206,6 |
| Total | 12923 | 47 |  |
|  |  |  |  |
| Newman-Keuls Multiple Comparison Test | Mean Diff | q | summary |
| MK-801 vs Control | -27,01 | 5,785 | ** |
| MK-801 vs Lurasidone (0.1) | -18,20 | 4,005 | * |
| MK-801 vs Lurasidone (0.5) | -16,00 | 3,521 | * |
| MK-801 vs Lurasidone (0.03) | -7,789 | 1,668 | ns |
| Lurasidone (0.03) vs Control | -19,22 | 4,012 | * |
| Lurasidone (0.03) vs Lurasidone (0.1) | -10,41 | 2,230 | ns |
| Lurasidone (0.03) vs Lurasidone (0.5) | -8,211 | --- | ns |
| Lurasidone (0.5) vs Control | -11,01 | 2,358 | ns |
| Lurasidone (0.5) vs Lurasidone (0.1) | -2,200 | --- | ns |
| Lurasidone (0.1) vs Control | -8,811 | --- | ns |

Lu AF21934+ Lurasidone

Main effects:

| Effects | Univariate Tests of Significance for Var1  Sigma-restricted parametrization  Effective hypothesis decomposition | | | | |
| --- | --- | --- | --- | --- | --- |
|  | SS | Degr. Of Freedom | MS | F | p |
| Intercept | 113125,9 | 1 | 113125,9 | 715,8156 | 0,000000 |
| LuAF21934 | 1754,3 | 1 | 1754,3 | 11,1006 | 0,002046 |
| Lurasidone | 1214,5 | 1 | 1214,5 | 7,6846 | 0,008863 |
| LuAF21934+ Lurasidone | 783,3 | 1 | 783,3 | 4,9561 | 0,032534 |
| Error | 5531,3 | 35 | 158 |  |  |

Post-hoc analysis

| Cell No. | Newman-Keuls test; variable Var 1  Approximate Probabilities for Post Hoc Tests  Error:Between MS=158,04, df=35,000 | | | | | |
| --- | --- | --- | --- | --- | --- | --- |
|  | LuAF21934 | Lurasidone | 1  46,1 | 2  48,3 | 3  50,556 | 4  70,7 |
| 1 | 0 | 0 |  | 0,701971 | 0,716644 | 0,000813 |
| 2 | 0 | 1 | 0,701971 |  | 0,694834 | 0,001192 |
| 3 | 1 | 0 | 0,716644 | 0,694834 |  | 0,001300 |
| 4 | 1 | 1 | 0,000813 | 0,001192 | 0,001300 |  |

Fig. 7 Spatial delayed alteration

Lu AF21934+ WAY100635

Main effects:

| Effects | Univariate Tests of Significance for Var1  Sigma-restricted parametrization  Effective hypothesis decomposition | | | | |
| --- | --- | --- | --- | --- | --- |
|  | SS | Degr. Of Freedom | MS | F | p |
| Intercept | 722,000 | 1 | 722,000 | 425,6 | 0,000000 |
| LuAF21934 | 24,500 | 1 | 24,500 | 14,4421 | 0,000716 |
| WAY100635 | 18,0 | 1 | 18,0 | 10,6105 | 0,002944 |
| LuAF21934+ WAY100635 | 8,0 | 1 | 8,0 | 4,7158 | 0,038514 |
| Error | 47,5 | 28 | 1,6964 |  |  |

Post-hoc analysis

| Cell No. | Newman-Keuls test; variable Var 1  Approximate Probabilities for Post Hoc Tests  Error:Between MS=1,6964, df=28,000 | | | | | |
| --- | --- | --- | --- | --- | --- | --- |
|  | LuAF21934 | WAY100635 | 1  4,1250 | 2  3,6250 | 3  6,8750 | 4  4,3750 |
| 1 | 0 | 0 |  | 0,449195 | 0,000756 | 0,704092 |
| 2 | 0 | 1 | 0,449195 |  | 0,000302 | 0,491318 |
| 3 | 1 | 0 | 0,000756 | 0,000302 |  | 0,000768 |
| 4 | 1 | 1 | 0,704092 | 0,491318 | 0,000768 |  |

Lu AF21934+ RS-8-OH-DPAT

Main effects:

| Effects | Univariate Tests of Significance for Var1  Sigma-restricted parametrization  Effective hypothesis decomposition | | | | |
| --- | --- | --- | --- | --- | --- |
|  | SS | Degr. Of Freedom | MS | F | p |
| Intercept | 712,5313 | 1 | 712,5313 | 316,0535 | 0,000000 |
| LuAF21934 | 26,2812 | 1 | 26,2812 | 11,6574 | 0,001969 |
| RS-8-OH-DPAT | 9,0313 | 1 | 9,0313 | 4,0059 | 0,055116 |
| LuAF21934+ RS-8-OH-DPAT | 30,0313 | 1 | 30,0313 | 13,3205 | 0,001066 |
| Error | 63,1250 | 28 | 2,2545 |  |  |

Post-hoc analysis

| Cell No. | Newman-Keuls test; variable Var 1  Approximate Probabilities for Post Hoc Tests  Error:Between MS=2,2545, df=28,000 | | | | | |
| --- | --- | --- | --- | --- | --- | --- |
|  | LuAF21934 | RS-8-OH-DPAT | 1  4,25 | 2  3,3750 | 3  4,1250 | 4  7,1250 |
| 1 | 0 | 0 |  | 0,483176 | 0,869062 | 0,000793 |
| 2 | 0 | 1 | 0,483176 |  | 0,326468 | 0,000300 |
| 3 | 1 | 0 | 0,869062 | 0,326468 |  | 0,001301 |
| 4 | 1 | 1 | 0,000793 | 0,000300 | 0,001301 |  |

Fig. 8 Microdialysis studies

The effect of Lu AF21934 (5 mg/kg) on the 5-HT release

Main effects:

| Effects | Repeated Measures Analysis of Variance (SER MK LU.sta)  Sigma-restricted parametrization  Effective hypothesis decomposition | | | | |
| --- | --- | --- | --- | --- | --- |
|  | SS | Degr. Of Freedom | MS | F | p |
| Intercept | 2252525 | 1 | 2252525 | 1781,369 | 0,000000 |
| traet | 203540 | 3 | 67847 | 53,655 | 0,000000 |
| Error | 21496 | 17 | 1264 |  |  |
| TIME | 53006 | 8 | 6626 | 25,001 | 0,000000 |
| TIME*treat | 52229 | 24 | 2176 | 8,212 | 0,000000 |
| Error | 36042 | 136 | 265 |  |  |

Post-hoc analysis

| Cell No. | Tukey HSD test; variable DV_1 (SER MK LU.sta)1  Approximate Probabilities for Post Hoc Tests  Error:Between MS=2,2545, df=28,000 | | | | |
| --- | --- | --- | --- | --- | --- |
|  | treat | 1  102,31 | 2  163,99 | 3  95,603 | 4  92,501 |
| 1 | sal |  | 0,000179 | 0,832985 | 0,652724 |
| 2 | MK-801 | 0,000179 |  | 0,000178 | 0,000178 |
| 3 | xxx | 0,832985 | 0,000178 |  | 0,979253 |
| 4 | MK+LuAF21934 | 0,652724 | 0,000178 | 0,979253 |  |

The effect of Lu AF21934 (5 mg/kg) on DA release

Main effects:

| Effects | Repeated Measures Analysis of Variance (SER MK LU.sta)  Sigma-restricted parametrization  Effective hypothesis decomposition | | | | |
| --- | --- | --- | --- | --- | --- |
|  | SS | Degr. Of Freedom | MS | F | p |
| Intercept | 4254128 | 1 | 4254128 | 3162,89 | 0,000000 |
| traet | 789681 | 3 | 263227 | 195,706 | 0,000000 |
| Error | 21520 | 16 | 1345 |  |  |
| TIME | 159451 | 8 | 19931 | 27,465 | 0,000000 |
| TIME*treat | 269281 | 24 | 11220 | 15,461 | 0,000000 |
| Error | 92890 | 128 | 726 |  |  |

Post-hoc analysis

| Cell No. | Tukey HSD test; variable DV_1 (SER MK LU.sta)1  Approximate Probabilities for Post Hoc Tests  Error:Between MS=2,2545, df=28,000 | | | | |
| --- | --- | --- | --- | --- | --- |
|  | treat | 1  97,483 | 2  261,58 | 3  145,66 | 4  116,59 |
| 1 | sal |  | 0,000185 | 0,000294 | 0,132596 |
| 2 | MK-801 | 0,000185 |  | 0,000185 | 0,000185 |
| 3 | xxx | 0,000294 | 0,000185 |  | 0,008472 |
| 4 | MK+LuAF21934 | 0,132596 | 0,000185 | 0,008472 |  |
